# Giant paleo-seafloor craters and mass wasting associated with magma-induced uplift of the upper crust

**DOI:** 10.1038/s41598-022-08205-0

**Published:** 2022-03-15

**Authors:** K. O. Omosanya, K. Duffaut, T. M. Alves, O. E. Eruteya, S. E. Johansen, N. Waldmann

**Affiliations:** 1 Department of Geoscience and Petroleum, Norwegian University of Science and Technology, Trondheim, Norway; 2 Oasisgeokonsult, 7052 Trondheim, Norway; 3 3D Seismic Lab, School of Earth and Environmental Sciences, Cardiff University, Main Building, Park Place, Cardiff, CF10 3AT UK; 4 Department of Earth Sciences, Geo-Energy/Reservoir Geology and Basin Analysis Group, University of Geneva, Geneva, Switzerland; 5 Dr Moses Strauss Department of Marine Geosciences, University of Haifa, Haifa, Israel

**Keywords:** Geodynamics, Geology, Geophysics, Tectonics, Volcanology

## Abstract

Giant seafloor craters are known along many a continental margin with recurrent mass-wasting deposits. However, the impact of breakup-related magmatism on the evolution of such craters is barely understood. Using high-quality geophysical datasets, this work examines the genetic relationship among the location of magmatic sills, forced folds and the formation of giant paleo-seafloor craters underneath an ancient mass-transport complex in the Møre and Vøring basins, offshore Norway. The data reveal that forced folding of near-seafloor strata occurred because of the intrusion of several interconnected magmatic sills. Estimates of 1-dimensional uplift based on well data show that uplift occurred due to the intrusion of magma in Upper Cretaceous to Lower Eocene strata. Our findings also prove that subsurface fluid plumbing associated with the magmatic sills was prolonged in time and led to the development of several vertical fluid flow conduits, some of which triggered mass wasting in Neogene to Recent times. The repeated vertical expulsion of subsurface fluids weakened the strata on the continental slope, thereby promoting mass wasting, the selective cannibalization of the paleo-seafloor, and the formation of elongated craters at the basal shear zone of the mass-transport complex. Significantly, the model presented here proves a close link between subsurface magmatic plumbing systems and mass wasting on continental margins.

## Introduction

### Giant craters and mass wasting along continental margins

Craters and erosional features on continental slopes and rises are often associated with mass-transport complexes in areas as diverse as the Gulf of Cadiz^1–3^, the Algerian Margin^4^, California^5^, Cascadia Margin^6,7^, Israel^8^ and Japan^9,10^. Often relating to the initial region of failure, in which crown scarps and headwall scarps may be preserved, isolated scars on the distal parts of continental margins may have diverse origins, as in the case of those identified the Gulf of Cadiz or Israel^3,11^. In other offshore regions, erosional features and seafloor scarps are clearly associated with water percolation through the continental slope and important neo-tectonic activity^6,12^. Thus, it is crucial to understand the origin of seafloor erosional features, or craters, wherever identified on bathymetric or geophysical data. Moreover, craters on continental margin with records of recurrent mass wasting may relate to the pre-disposing factors that led to past submarine slope instability, or may lead to future instability processes we may be unaware of.

On the mid-Norwegian margin gigantic craters beneath submarine landslides, otherwise referred to as evacuation structures, have been previously reported in the literature^13,14^. Coincidentally, this margin is characterized by its extensive networks of magmatic sills and hydrothermal vents^15,16^. About 2000–3000 hydrothermal vent complexes were developed in Cenozoic strata of the Møre and the Vøring basins following the widespread intrusion of magma during the opening of the Norwegian and Greenland Seas^15^. On the Modgunn Arch alone, about 125 sills and 85 hydrothermal vent complexes were identified and mapped by^17^, several of which have been repeatedly reutilized after they were formed in the Paleocene-Eocene. Consequently^13,14^ proposed that seafloor craters and other incisional features were formed due to fluid migration from beneath ooze intervals, or by the liquefaction of oozes in response to loading by younger submarine landslides. Previously^13^ had described such craters as “evacuation structures’’ and mainly attributed their evolution to loading and fluid expulsion from Oligocene–Miocene ooze intervals. However, direct correlations between the magmatic sills mentioned above, and the giant craters studied by^14^ and^13^, are not known for other parts of the Norwegian Sea.

In this work, we investigate the primary model proposed by^14^ within the context of localized uplift during Eocene magmatism. To achieve our aims, we use multiple seismic reflection data, regional 2D seismic lines, and well data to explore the correlation between several km-scale, elongated craters along the basal shear zones of an ancient mass-transport deposit (MTC), and underlying networks of magmatic sill complexes, paleo-highs, and fluid plumbing elements. The term mass-transport complex (MTC), as defined in this work, encompasses distinct deepwater sedimentary packages such as submarine landslides and debris flow deposits, or debrites. Importantly, the high-quality dataset analyzed here, imaging the Storegga area and the Modgunn Arch, provides an exceptional opportunity to reassess the geometry of the craters formed at the base of the MTC of interest, and allow the proposition of a new conceptual model to explain their formation. This study provides unique evidence for the mechanisms driving the development of such large craters along the North Atlantic Igneous Province and has wider implications for our understanding of similar craters on other continental margins.

## Geologic setting

**Figure 1:**
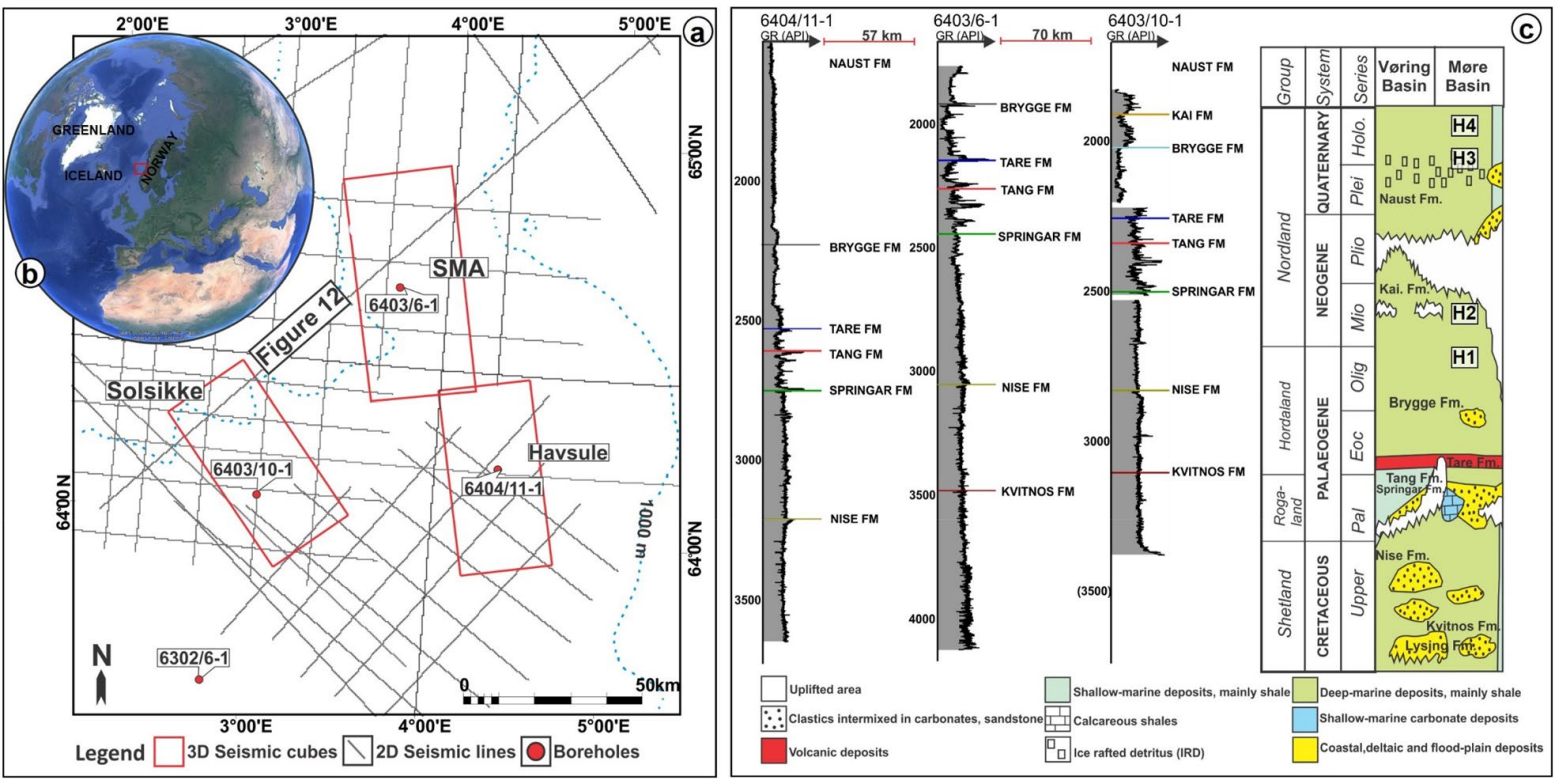
(**a**) Map showing the location of the 3 three-dimensional (3-D) and two-dimensional (2-D) seismic reflection data, plus the well data used in this study (**b**) Inset shows the location of the study area in the context of the Norwegian continental margin. The red box outlines the study area. (**c**) Well correlation panel between wells 6404/11-1, 6403/6-1, and 6403/10-1 and a simplified stratigraphic column for the study area. This study focuses on the stratigraphic interval spanning from the Brygge to the Naust formations. Included in the column are the main representative lithologies and the regional lithostratigraphy of the Vøring and Møre basins.

The study area is part of the Norwegian margin (Fig. 1), a divergent continental margin on which several Holocene submarine landslides such as the Storegga, Møre and Tampen Slides are located^18^. The tectonic evolution of the Norwegian margin records a series of post-Caledonian rift phases that culminated with the onset of seafloor spreading at about 56 Ma^19^. Main rifting episodes affecting the margin are dated back to the Early-Middle Devonian, Carboniferous, Late Permian-Early Triassic, Jurassic-Earliest Cretaceous and the latest Cretaceous-Paleocene^20^. The main phase of infill of the Møre and Vøring basins occurred in the Cretaceous during a period of relative tectonic quiescence^21^. Exceptionally thick Cretaceous sequences were deposited because of the combined action of thermal subsidence, lithospheric deformation due to loading, and compaction of underlying pre-Cretaceous strata^21,22^. In the Møre and Vøring basins, Cretaceous deposits can locally reach 13 km in thickness^23^.

The Møre and Vøring basins were subjected to complex tectonism, differential sedimentation and erosion during the Cenozoic^22,24^. They became largely fed by sediment sourced from Fennoscandia and the inner parts of the mid-Norwegian shelf between the Oligocene and the Early Pliocene. In addition, the mid-Norwegian margin experienced multiple compressional events in the Miocene that promoted the development of domes, arches and elongated antiforms^20,25^. A large portion of the deposits resulting from these tectonic events consist of biogenic oozes^13^, now included in the Brygge, Kai and the Molo formations, comprising a significant volume of strata in the youngest Naust Formation. These sediments were mainly driven by the development and subsequent fluctuation of the Fennoscandian Ice Sheet through time^24,26^. Submarine landslides developed in the Pliocene–Pleistocene Naust Formation consist of biogenic oozes, contourites, hemipelagites and glaciogenic sediments^26,27^.

Submarine landslides along the mid-Norwegian Sea were triggered by sediment redistributed through the waxing and waning of the Fennoscandian Ice Sheet, seismic loading, fault activity, and gas hydrate dissociation^16,28^. The development of the first submarine landslides probably coincided with glacial intensification in the circum-Atlantic region at approximately 2.7 Ma^29^. Ice sheets reached the Norwegian shelf edge for the first time at about 1.1 Ma^30^. Large-scale submarine landslides were initiated in the region after the onset of recurrent glaciations on the continental shelf, from ca. 0.5 Ma onwards, peaking at 8.2–8.1 ka with the development of the Storegga Slide, which reaches a total area of 95,000 km^2^ and a run out distance of 800 km^27,31^.

## Stratigraphic framework

The different stratigraphic formations recognised on the Norwegian margin, as drilled by the exploration wells shown in Fig. 1c, include the Cretaceous-Paleocene Nise, Springar, Tang, Tare, Brygge, Kai and Naust Formations. The lowermost Springar Formation consists of greyish-green claystones interbedded with stringers of carbonate and sandstone^32^. Campanian to Maastrichtian in age, this formation was deposited in an open marine environment. On top of the Springar Formation lies the Paleogene Tang Formation, a sequence of dark-grey to brown claystones with minor sandstone and limestone intervals^32^. Strata in the Tang Formation were deposited in a deep-marine environment. The base of the Tare Formation is defined by an increase in tuff content and comprises dark-grey, green, or brown claystones with thin sandstone stringers and a variable content of tuffaceous material^32^. The overlying Lower Eocene to Lower Miocene Brygge Formation^32,33^ consists of claystone units with biogenic oozes, stringers of sandstone, siltstone, limestone and marl^32,34^. Pyrite, glauconite and shell fragments are present in the sandstones, hence pointing to a deep (hemipelagic) marine environment^35^. In the South Modgunn area, the Brygge Formation was the first unit deposited after continental breakup; it is highly deformed by two groups of polygonal faults and radial faults formed above hydrothermal vents^16^. Alternating claystone, siltstone and sandstone with limestone stringers occur in the overlying Kai Formation. The Kai Formation was deposited from the Middle Miocene to the Pliocene in a marine environment of varying water depths^32,35^. The youngest formation in the study area is the Naust Formation, comprising claystone, siltstone and sand with occasional coarse siliciclastics in its upper part. The Naust Formation is Upper Pliocene in age and was deposited in a marine environment^32^. A transition to glaciomarine environments is recorded in its upper part, but such a transition is poorly documented by exploration wells^24^.

## Mass-transport complex (MTC X)

**Figure 2:**
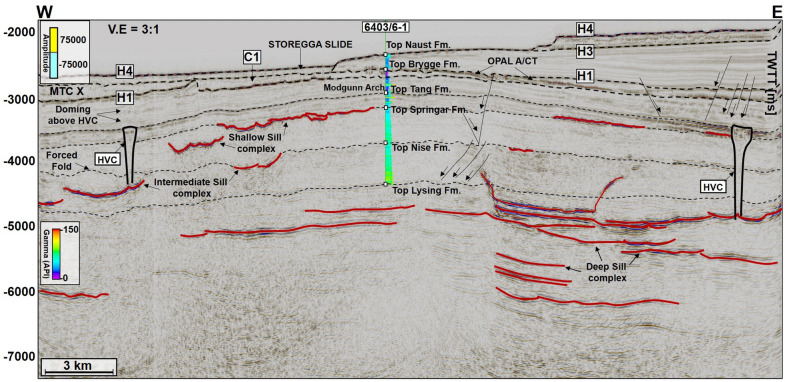
W–E seismic reflection profile showing the stratigraphic framework of the MC3D-MGS2002-FULL-OFFSET_3D_FM_TVFGC (SMA) seismic volume. The surface of interest is horizon H1, which corresponds to the Top Brygge Formation in well 6403/6-1. The strata underlying the study interval are often intruded by magmatic sills. In addition, hydrothermal vent complexes are also interpreted in the proximity of some of the magmatic sills. N.B: The uninterpreted seismic profile is provided in the appendix.

**Figure 3:**
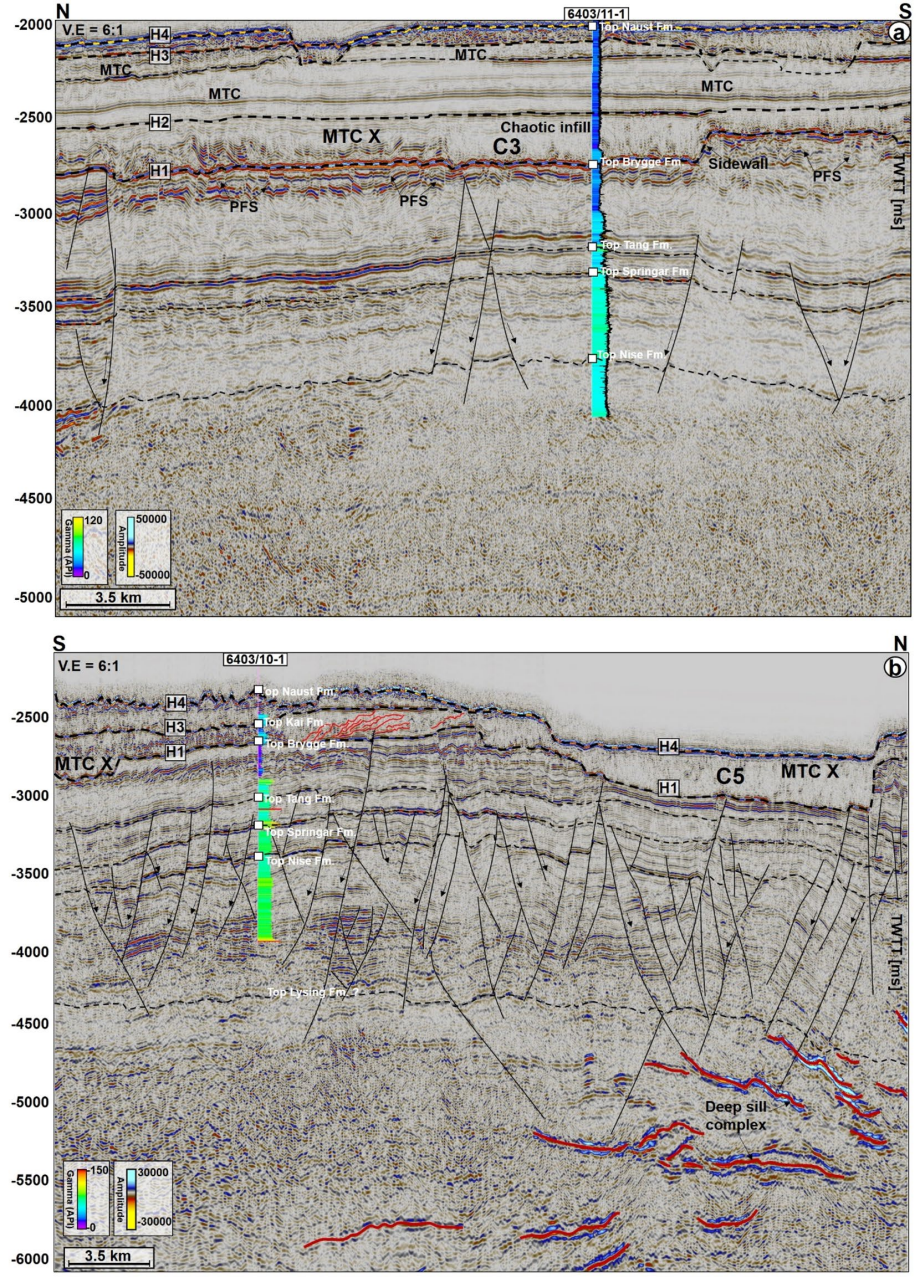
(**a**) N–S seismic profiles through the MC3D-RHD99_3D_FM_TVFGC survey (Havsule) and, (**b**) S–N arbitrary line through the NH0003-FULL_3D_FM_TVFGC survey (Solsikke) and well 6403/10-1. Both sections show the stratigraphic framework of the study area plus the link between craters, faults and underlying magmatic sill complexes. N.B: Uninterpreted seismic profiles are provided in the appendix. The location of the seismic sections is shown in Fig. 4.

**Figure 4:**
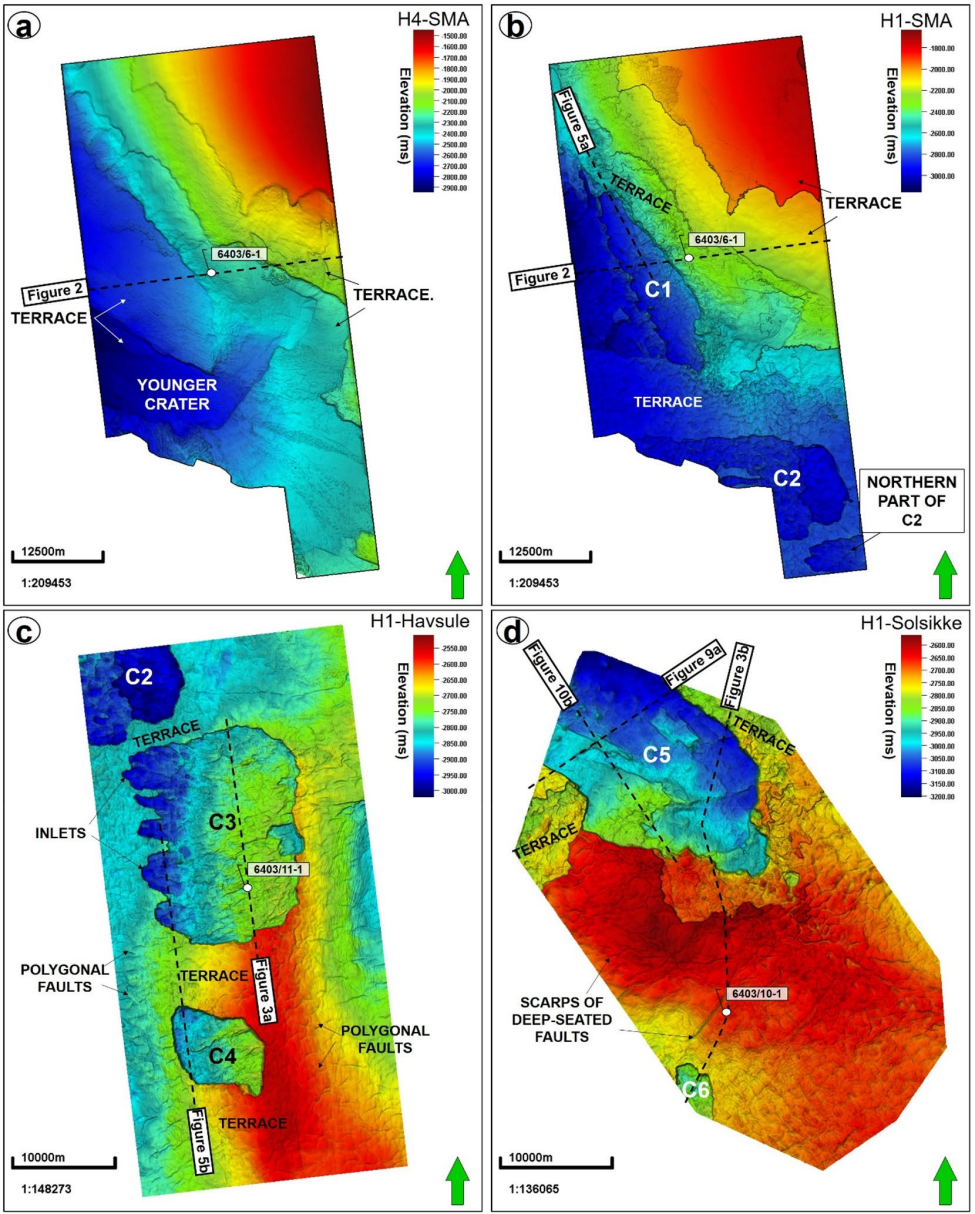
Structural maps of (**a**) the seafloor (horizon H4) in the SMA area, and horizon H1, i.e., the Top Brygge Formation in the (**b**) SMA (**c**) Havsule and (**d**) Solsikke areas. Also shown on the maps are Craters C1-C6 and the location of some of the interpreted seismic profiles.

**Figure 5:**
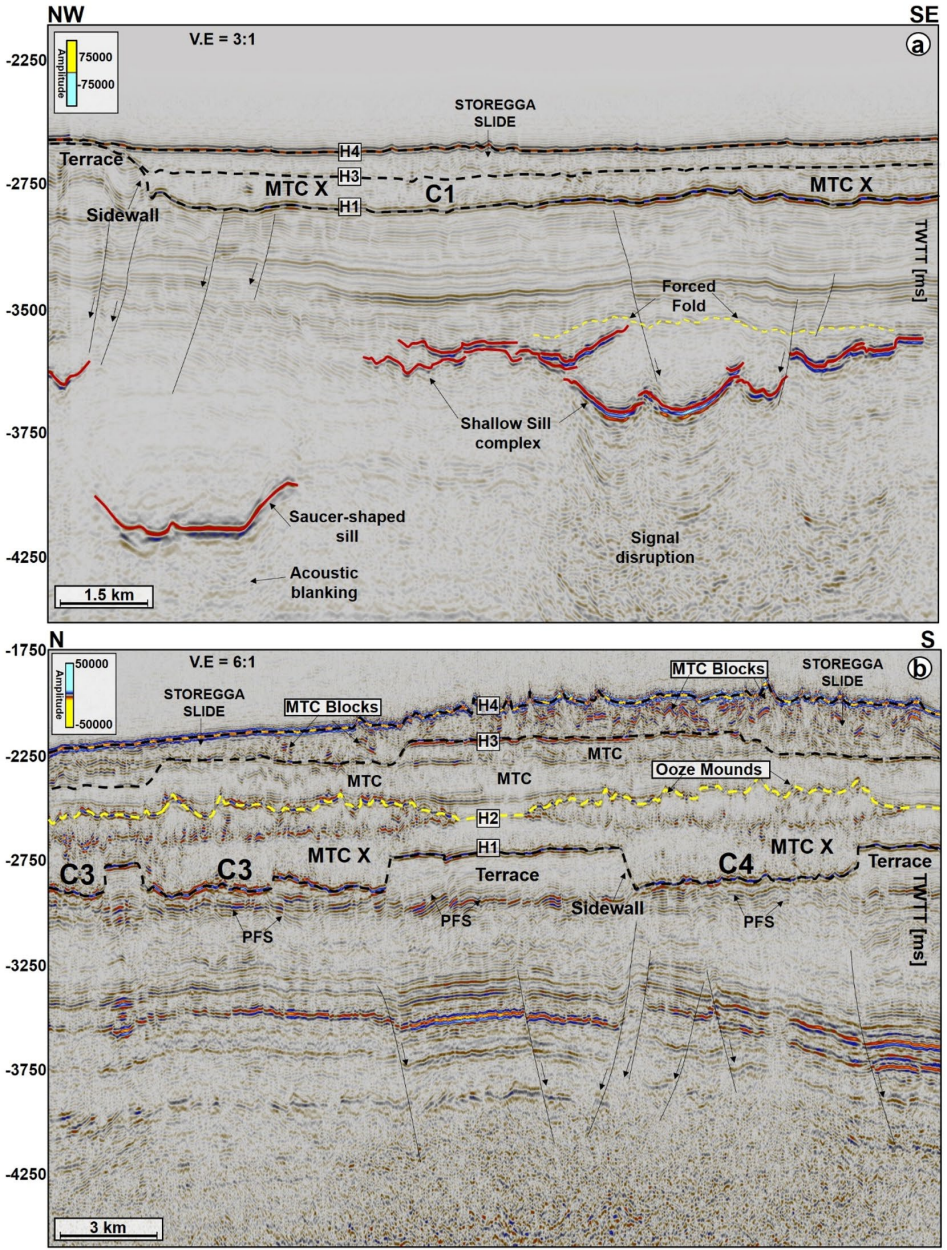
Seismic profiles showing the basal shear zone (BSZ) configuration of MTC X in the (**a**) SMA and (**b**) Havsule areas. The BSZ of the MTC is characterized by its rugged topography. Here, marked topographic variations on the BSZ are caused by craters, their flanking terraces (flats), and the sidewalls that connect the craters to the terraces. All these BSZ features are notably underlain by magmatic sills in the SMA area and by polygonal faults in the Havsule area. The infill of the craters varies considerably from chaotic, low-amplitude strata (reflecting debris-flow deposits in the SMA area) to a mixture of moderate to high-amplitude strata in the Havsule area. N.B: Uninterpreted seismic profiles are provided in the appendix.

**Figure 6:**
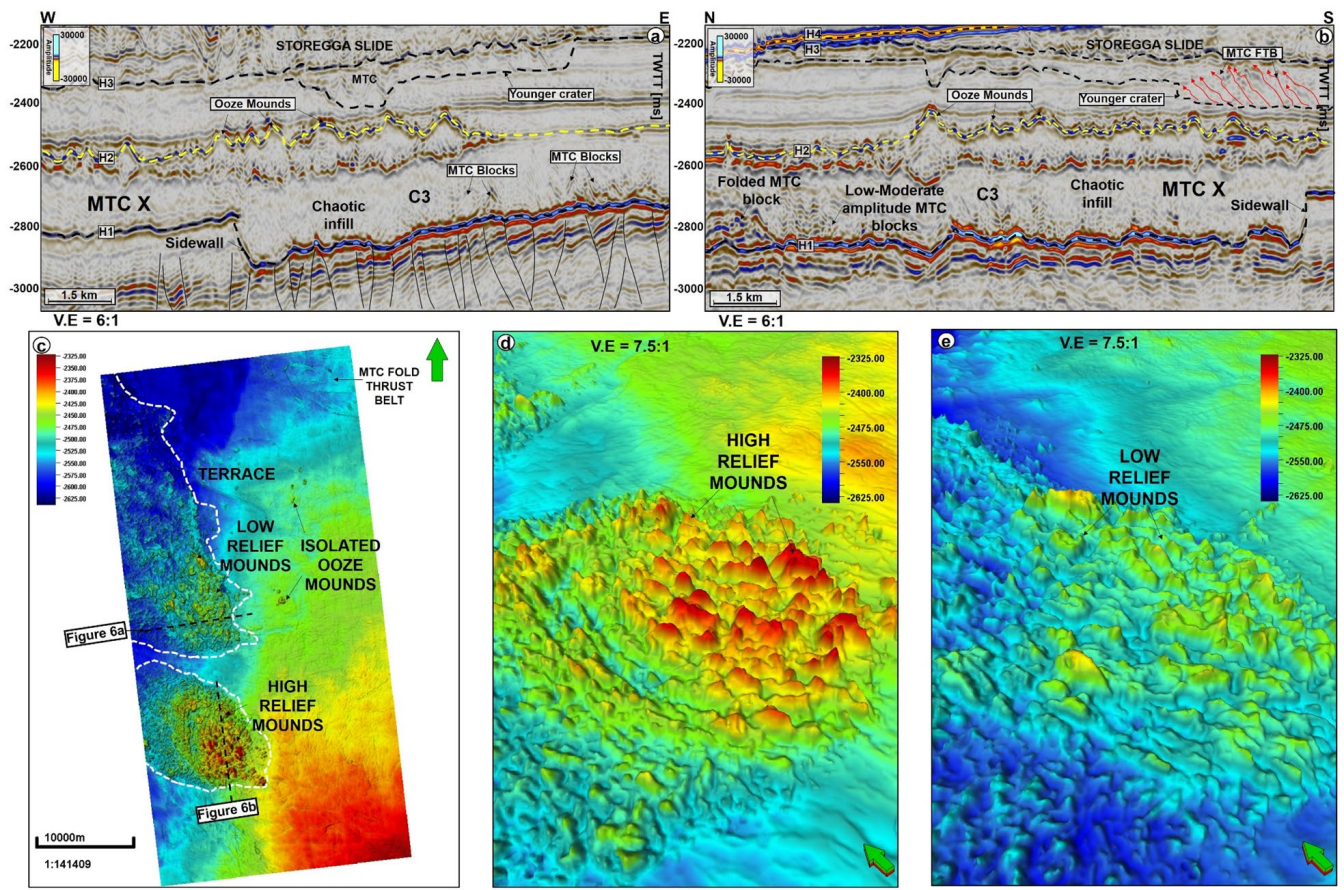
(**a**,**b**) Seismic profiles showing the distinct infill of C3 and the unusual presence of ooze mounds above this crater. Craters C3 and C4 in the Havsule area are distinctly underlain by polygonal faults and their tops reveal high to low relief ooze mounds. Where high relief ooze mounds are interpreted, the overburden is immediately characterized by younger craters. In addition to the chaotic infill of the craters, moderate to high amplitude slide blocks are also found within the craters. N.B: Uninterpreted seismic profiles are provided in the appendix. (**c**) Structural map of horizon H1 showing the ooze mounds distributed into two distinct domains. Isolated mounds are also found on the eastern part of MTC X. (**d**,**e**) 3D view of the two ooze-mound domains.

**Figure 7:**
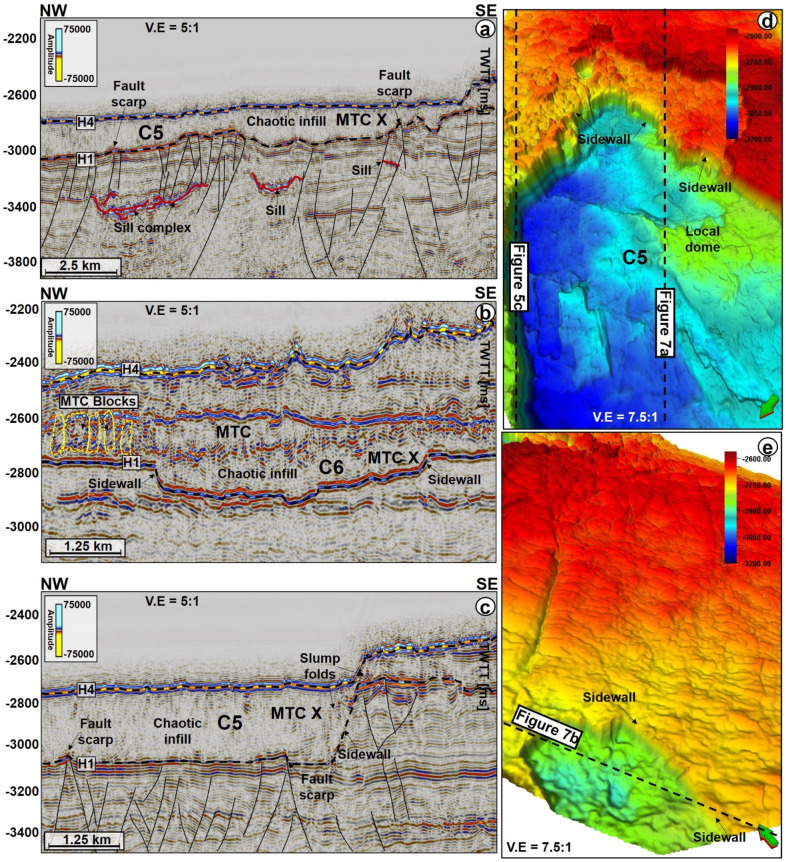
(**a**–**c**) Seismic profiles showing the infill types within C5 and C6 in the Solsikke area. Magmatic sills or faults are found in the places where the BSZ is arcuate or uplifted. N.B: Uninterpreted seismic profiles are provided in the appendix. (**d**,**e**) 3D perspective of the location of the seismic profiles in (**a**,**c**) and the differences in the architecture of the BSZ of MTC X. In addition, C6 is capped by a MTC comprising a heterogenous mixture of slide (or rafted) blocks and chaotic reflections.

MTC X is the main mass-transport complex of interest to this work and occurs in Cenozoic strata of the Møre and Vøring basins. In seismic data (Figs. 2, 3, 4, 5, 6, 7), the base of MTC X correlates with Horizon H1, which also marks the top of the Brygge Formation in wells 6404/11-1, 6403/6-1 and 6403/10-1 (Figs. 1c, 2 and 3). Horizon H1 is a high-amplitude negative, continuous, seismic reflection that coincides with a fossilized Opal A/Opal CT boundary (Fig. 2). See also^13,36^. Internally, MTC X comprises low- to moderate-amplitude reflections that are often intercalated with chaotic reflections (Figs. 2, 3, 4, 5, 6, 7). In the SMA and Solsikke areas, most of MTC X is composed of seismically homogeneous to chaotic deposits with low seismic amplitude (Figs. 2 and 3b). In the Havsule area MTC X comprises folded MTC blocks and low-moderate amplitude blocks (Figs. 3a, 6a,b). Common features at the base of MTC X include extensional faults extending into deeper strata (Figs. 2 and 3), polygonal faults (Figs. 3b, 4 and 5b), and discrete craters (Fig. 4).

**Figure 8:**
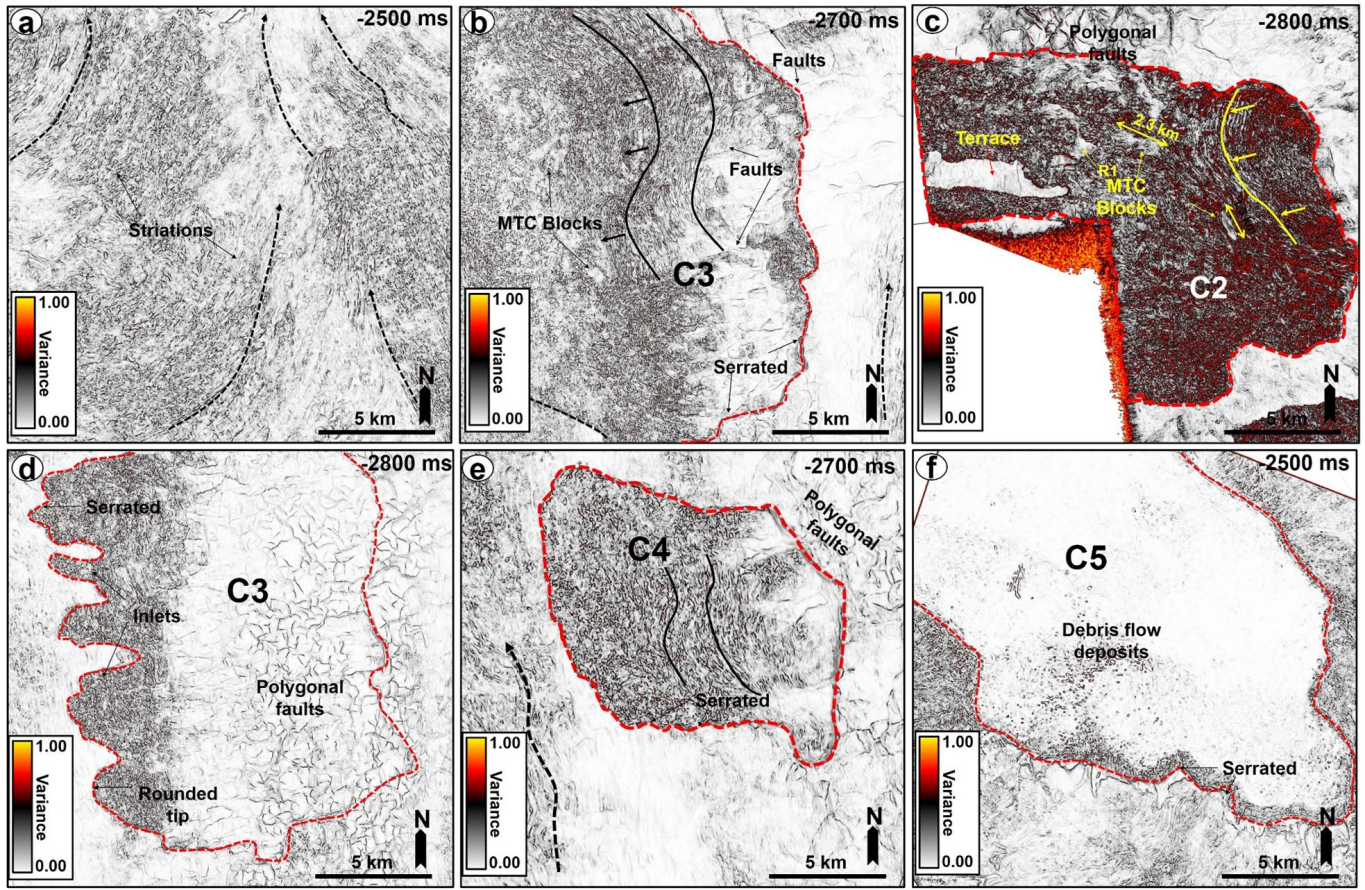
Variance maps showing orientation of striations, an outline of the craters, and compositional variations in strata filling the craters. (**a**) N- to NW oriented striations at the base of MTC X in the Solsikke area. The craters are characterized by chaotic dark, grey, and heterogeneous reflections on the variance maps, whereas the terraces are white to reflection-free sections that contrast sharply with the adjacent craters. The variance maps also reveal that the internal and external geometries of the craters include (**b**) slide blocks from the SMA area (**c**–**e**) Serrated distal inlets with slide blocks, faults, and slump folds in the Havsule area and (**f**) Chaotic reflections akin to debris-flow deposits within C5. *R1- Rafted block 1.*

The top of MTC X is variable across the three areas analyzed in this work. In the SMA area, the top of MTC X corresponds to Horizon H3 (Top Kai Formation), which also coincides with the base of the youngest MTC in the area i.e., the Storegga Slide (Figs. 2 and 5a). In the Havsule area, the top of MTC X coincides with Horizon H2, itself marking the top of the ooze mounds. Here, the top of the ooze mounds is incised by overlying, younger MTCs (e.g., Fig. 5b). In addition, the upper part of MTC X in the Havsule area is dominated by lensoid- and diapir-like features reaching 100–120 ms TWTT (110 m to 132 m) in height (Figs. 5b, 6a,b). Such features comprise ooze mounds (^13,14^) and, in seismic data, are characterized by significant relief at their basal and top surfaces (Fig. 6). Ooze mounds in the Havsule area are spatially distributed in two domains (Fig. 6c). The first domain shows high-relief (> 100 ms) mounds where MTC X is characterized by purely homogeneous reflections or a chaotic fill (Fig. 6a,d). A second domain with much smaller mounds (< 100 ms) is observed where the internal composition of MTC X is heterogeneous (Fig. 6b,e). In the Solsikke area, the top of MTC X coincides with Horizon H3 away from crater C5 and in the southern part of the survey (Figs. 3b and 7b). In the northern part of the Solsikke area, where C5 is present, the top of MTC X correlates with Horizon H4 (Top Naust Formation; Figs. 3b, 7a,c). MTC X reveals a predominant N to NNW direction of transport based on the orientation of striations interpreted in Fig. 8a,b.

## Craters beneath MTC X

**Figure 9:**
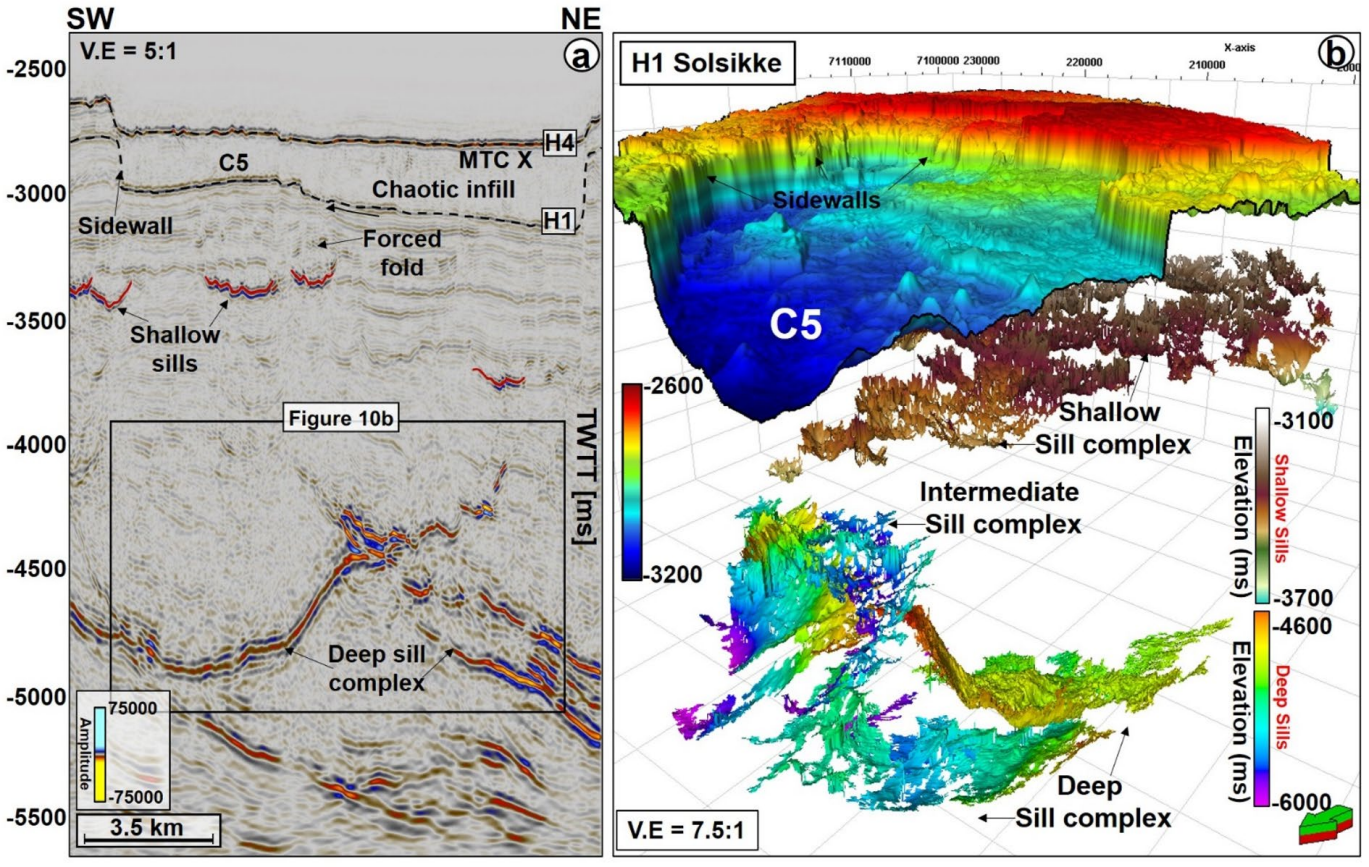
(**a**) SW-NE seismic profile showing crater C5 and underlying magmatic sills at a depth between 3300 ms and 5500 ms TWTT. Most of the shallow sills in the Solsikke area are interconnected saucer-shaped sills. N.B: An uninterpreted seismic profile is provided in the appendix. (**b**) 3D view showing the spatial relationship between C5 and underlying magmatic sills. In the study area, the interpreted magmatic sills are classified into shallow sills at depths < 4000 ms TWTT, intermediate sills occurring at a depth of 4000–5000 ms TWTT, and deeper sills occurring at depth > 5000 ms TWTT.

Craters in the basal shear zone of MTC X represent negative topographic features that are associated with the incision of paleo-seafloor strata by failed sediment masses (Figs. 2, 3, 4, 5, 6, 7, 8, 9). Seven craters (C1-C7) were interpreted in this study (Fig. 4), two in each of the investigated areas, i.e., the SMA (C1 and C2), the Havsule area (C3 and C4), and the Solsikke area (C5 and C6). C7 is the last crater located on the regional 2D seismic profile connecting the Solsikke and the SMA areas (Fig. 13). Furthermore, C2 in Havsule overlaps with the SMA area (Fig. 4b,c). Two distinct types of erosional features occur in the distal part of C3 (Figs. 4c and 5b). The first type comprises inlets, irregular bay-like features at the base of MTC X that are circular to oval in shape (Figs. 4c and 5b). Inlets only occur in C3. The second geomorphic structures are terraces or peninsula-like protrusions on the paleo-seafloor that flank all the craters and the inlets in C3 (Figs. 1c, 4 and 5). These terraces (or flats) are separated from the craters by steep sidewalls (Figs. 2, 3, 5, 6a,b), being relatively high, undeformed, continuous sections of the basal shear zone of MTC X (Figs. 1c, 4 and 5). Sidewalls of the craters at the base of MTC X are generally sub-vertical (Figs. 2, 3, 4, 5, 6, 7), and occasionally serrated in map view (Figs. 4c, 8d,f).

Internally, the craters reveal striations (Fig. 8a,b), imbrications (Fig. 8b), faults (Figs. 4d, 7a,c,d, 8b), MTC blocks (Figs. 6a,b, 8c,d), polygonally-faulted strata (Figs. 3a, 4c, and 8d) and debris flow deposits (Figs. 5, 7b, 8). In the SMA area, MTC blocks within the crater can reach up to 2.3 km in length, and a height between 163 and 197 ms (179 to 217 m); see block R1 in Fig. 8c. Imbricated strata and slump folds in craters C3 and C4 (SMA and Havsule areas) strike in a N–S direction, suggesting an E-W direction of local transport for the mobilized material (see Figs. 8b,c,e). In contrast to the craters at Havsule, those at Solsikke are dominated by chaotic to homogeneous seismic facies reflecting the presence of debris-flow deposits (Figs. 7 and 8f). Apart from the compositional variability displayed by the craters’ sediment fills, from east to west, all craters have boundaries that are abrupt and uniquely differentiated from adjacent transparent, homogeneous strata on variance maps (Fig. 8b–f). In parallel, the craters’ boundaries are regularly offset by extensional faults (Fig. 8b).

## Magmatic sills, forced folds, and fluid-flow structures associated with craters

**Figure 10:**
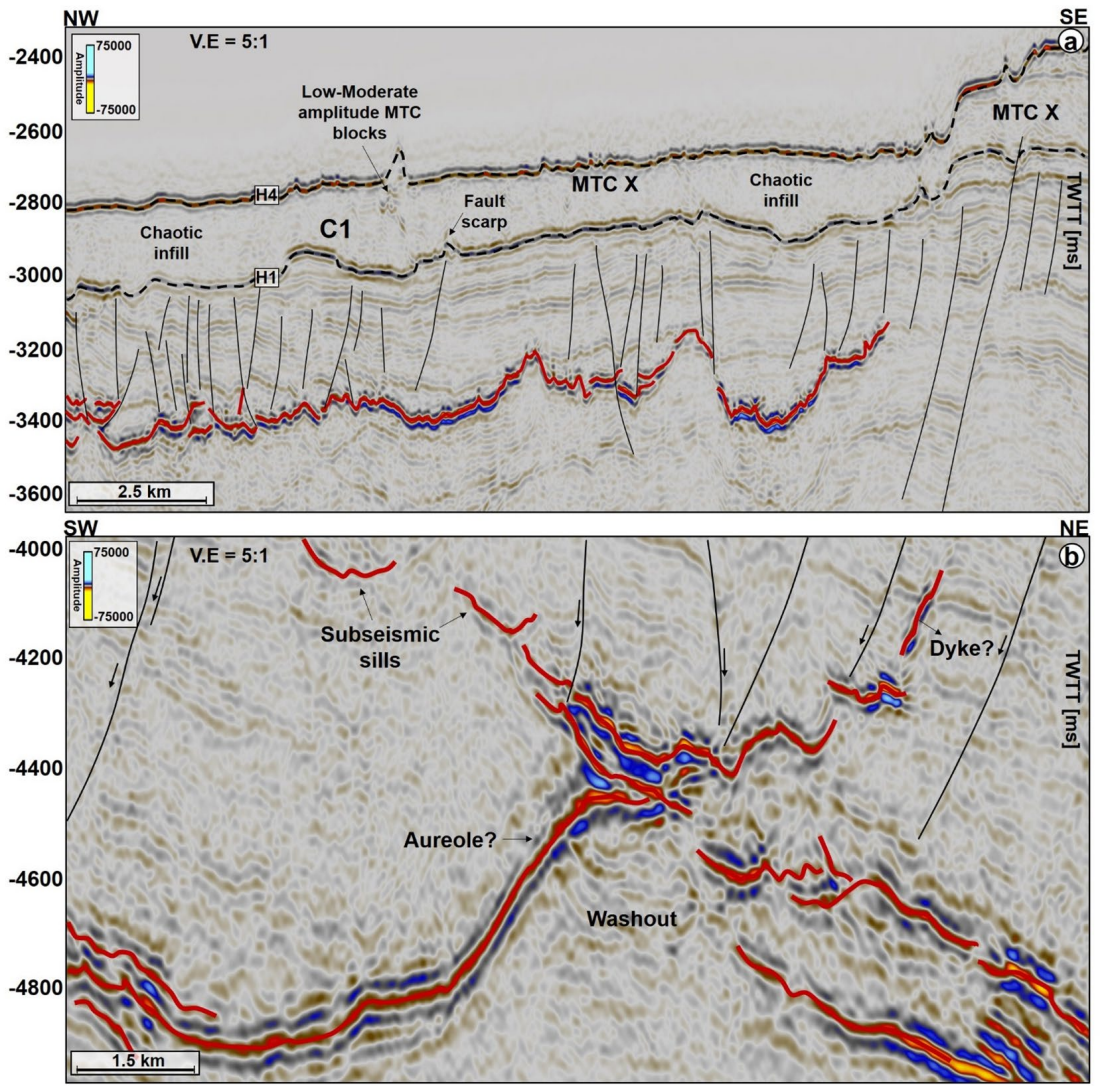
(**a**) NW–SE arbitary line showing several interconnected sills under C1 in the SMA area. (**b**) Zoomed-in image of the deeper sill complex in Fig. 9a. Deeper in the succession, some of the sills include low-moderate amplitude reflections, which are interpreted as thin magmatic sills below minimum seismic resolution. The amplitude washout zones around the sills are also interpreted as contact aureoles between the sills and their host strata. N.B: Uninterpreted seismic profiles are provided in the appendix.

**Figure 11:**
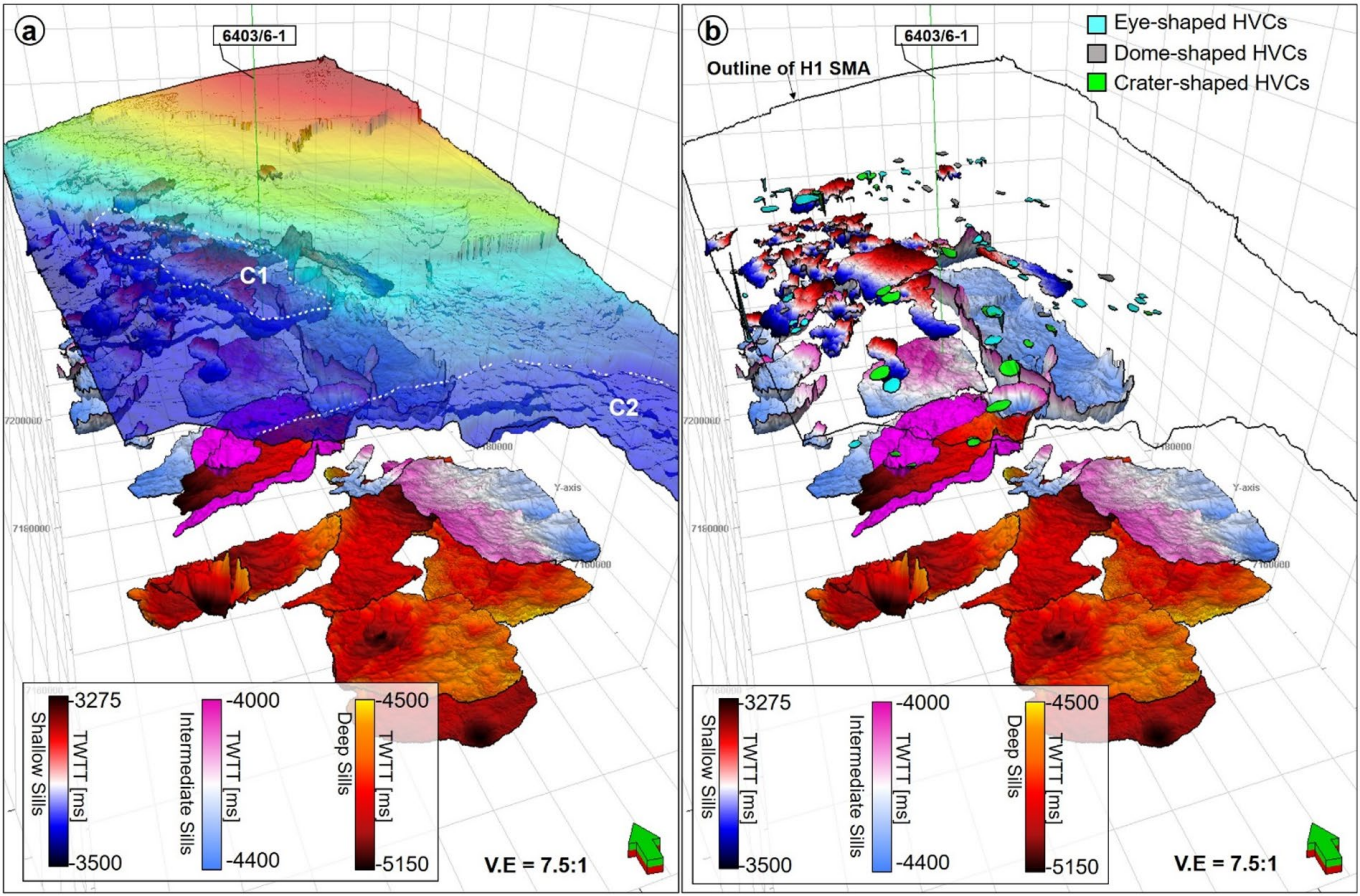
3D diagram highlighting the observation that the areas where craters are interpreted in the SMA area are also underlain by magmatic sills. (**a**) Structural map of horizon, H1 displayed with a 30% transparency. The spatial extent of the craters is marked by the white dashed polygons (**b**) Above the sills, different types of hydrothermal vent complexes (HVCs) are interpreted. These include eye-, dome- and crater-shaped HVCs.

**Figure 12:**
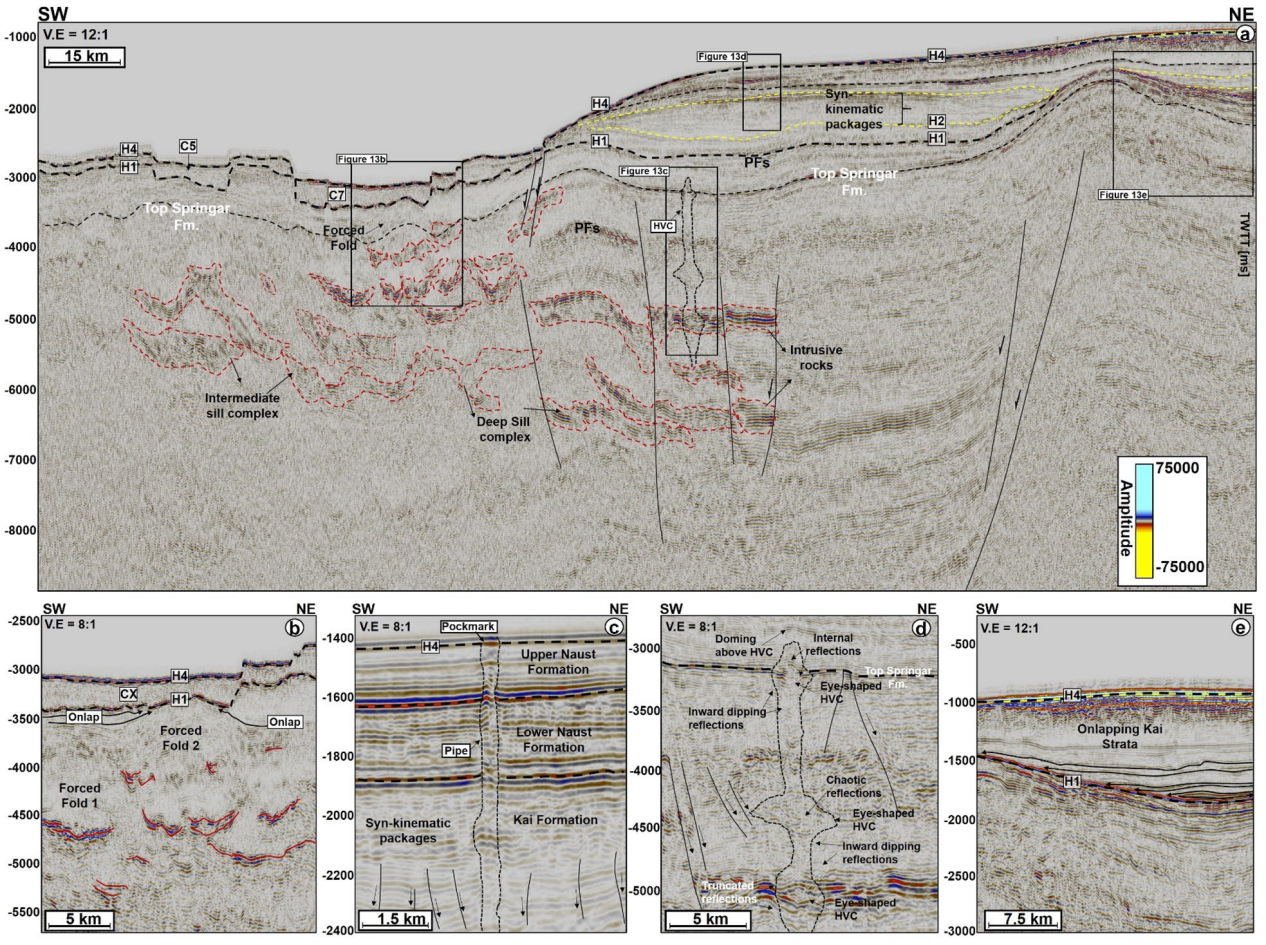
(**a**) Regional 2D seismic line showing forced folds and magmatic intrusions in the study area. The seismic line runs between the Solsikke and SMA areas. In addition, the figure shows evidence for (**b**) local uplift caused by magmatic sill emplacement under craters, and (**c**) an example of a vertical fluid-flow structure within the Naust Formation, revealing that vertical fluid seepage is a common phenomenon on the modern seafloor. (**d**) Example of a hydrothermal vent complex below a crater, a character providing evidence for protracted fluid flow in the study area. (**e**) Regional uplift related to basin evolution and dynamics. Onlapping reflections above the Brygge Formation show that the region was uplifted during the Miocene.

**Figure 13:**
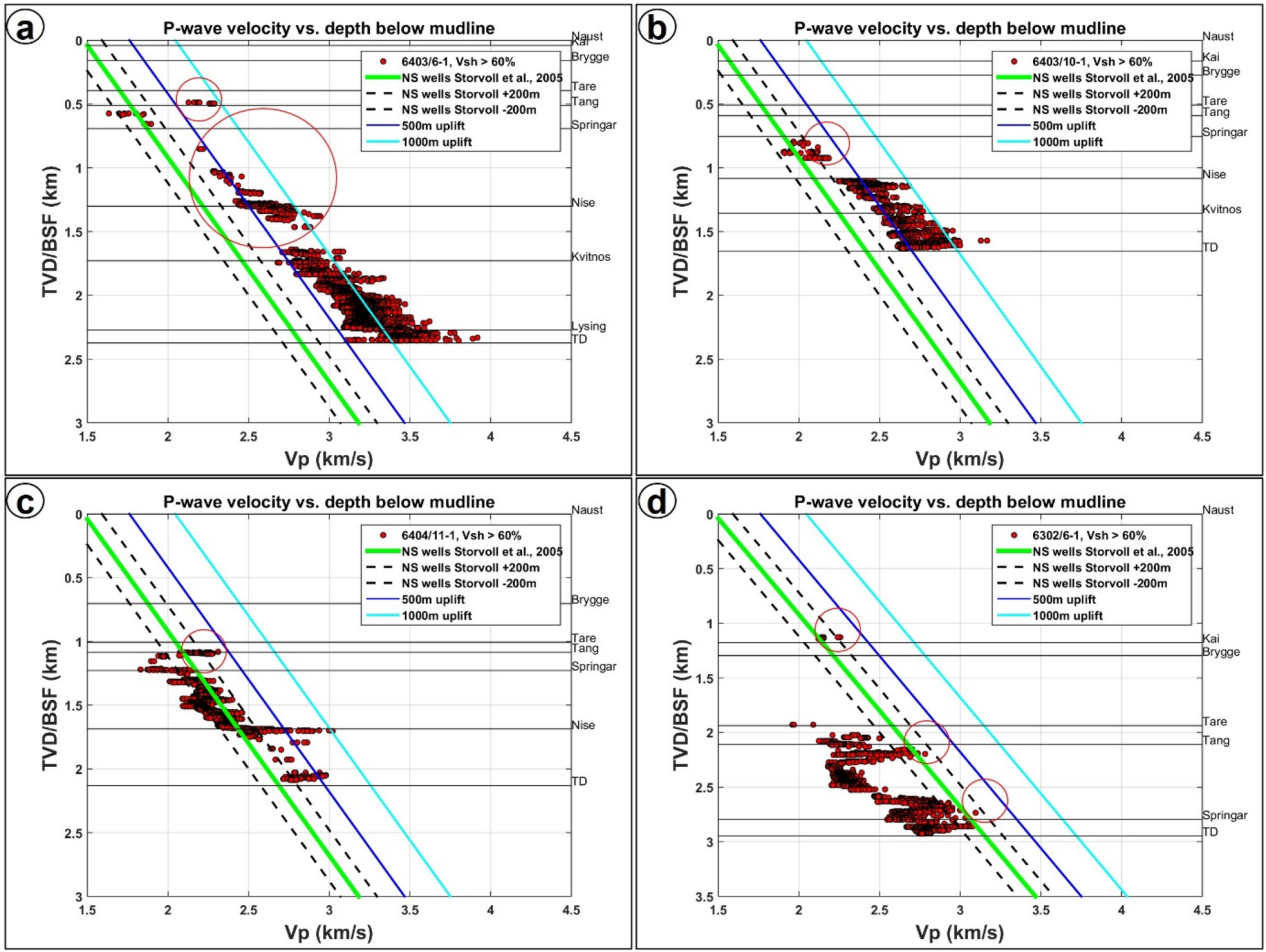
1D uplift cross plots for wells (**a**) 6403/6-1 (**b**) 6403/10-1 (**c**) 6404/11-1 and (**d**) 6302/6-1. Only well 6403/6-1 shows evidence of uplift and about 400 m to 700 m ± 200 m net erosion within the Springar Formation. Above the Springar Formation, about 750 m to 1000 m uplift is recorded within the Tare Formation. The first three wellbores are in areas where several magmatic sills have been interpreted while 6302/6-1 is outside the study area and not affected magmatic intrusions. N.B: The red circles highlight uplift at Springar, Tang and Kai levels.

Magmatic sills are interpreted beneath all the craters in the SMA and Solsikke areas (Figs. 2, 3b, 5a, 7a, 9, 10, 11 and 12a). In seismic data, magmatic sills are saucer-shaped high-amplitude reflections (Figs. 7a, 9a, and 10a) that are regularly interconnected to form complexes (Figs. 2, 5a and 13a). Based on their depth of occurrence, the sills can be classified into three groups: deep, intermediate, and shallow (Figs. 7, 10b, and 11). The deeper sills form an interconnected complex at depths greater than 5,000 ms TWTT (Figs. 9, 10, 11). At such a depth, the geometry of sills reach tabular to transgressive forms (Fig. 3). Intermediate magmatic sills occur at depths of 4,000 ms-5,000 ms TWTT, whereas the shallow sills occur above 4,000 ms TWTT (Figs. 9b and 11). An obvious consequence of magma intrusion in the study area was the generation of forced folds in the strata below the craters (Figs. 7a,d, 9a and 12). Sills in the Solsikke area occur below C5 and show mild uplift of strata above them, as indicated by the onlapping reflections above the sills (Fig. 5). In addition, an interconnected sill complex with two forced folds in its upper part is interpreted between the Solsikke and SMA areas (Figs. 12a,b). At the top of these two forced folds are onlapping seismic reflections that mark the timing of magmatic emplacement in the area; Early Eocene^37,38^. These onlapping reflections indicate that folds grew due to the intrusion of underlying sills, resulting in the formation of bathymetric highs that were onlapped by syn-kinematic deposits^39^.

In addition to the magmatic sills, hydrothermal vent complexes (HVCs) are also common in the study area. These HVCs are mostly found in the SMA and Solsikke areas (Figs. 2, 11a and 12). HVCs in the SMA area have dome-, eye-, and crater-shaped tops (Fig. 11b). The majority of these HVCs have summits in the Springar to the Tare formations (Fig. 2), a character suggesting an Early Eocene age for the bulk of the hydrothermal venting. An unusual geometry of vertical stack of eye-shaped vents can also be interpreted (Fig. 12a,d). This type of geometries is common within reutilized HVCs as previously documented in the More and Vøring basins by^17,40^. Evidence for younger Holocene fluid plumbing is provided by a subvertical fluid-escape pipe between the Solsikke and SMA areas (Fig. 12a,c). The top of the pipe corresponds to the top Naust Formation, or seafloor reflection, indicating recent fluid escape. In the Havsule area, seismically resolved magmatic sills are not found. This does not mean they are not present, as they could be of a thickness below seismic resolution, i.e., comprising ‘sub-seismic’ sills. However, the craters in the Havsule area are dominantly underlain by polygonal fault systems (Figs. 5b, 6a,b). These polygonal faults are valid proxies for relict episodes of fluid flow during the Upper Eocene to Miocene time-interval.

## Net erosion estimates from p-wave velocity variations

Estimates of net erosion and uplift based on variations in P-wave velocities at the four studied wells is provided for the Springar, Tang, Tare and Brygge formations (Fig. 13a–d). From well 6403/6-1 (SMA), only the Tare Formation shows evidence for uplift, which reaches significant values of 750 m to 1000 m ± 200 m (Fig. 13a). Estimated net erosion from the trend line is about 800 m ± 200 m, with a maximum of ~ 1100 m ± 200 m (Fig. 12a). The underlying Tang Formation shows no evidence for erosion and uplift. Net erosion for the upper of the Springar Formation is estimated at about 300 m to 700 m ± 200 m, with a minimum of 400 m ± 200 m and a maximum of 1000 m ± 200 m (Fig. 12a). Wells 6403/10–1 and 6404/11–1 show no evidence for erosion and strata uplift above the Springar Formation (Fig. 12b,c). However, about 250 m ± 200 m of uplift is estimated for the Springar Formation based on data from well 6403/10–1. This well, located outside the region where no magmatic intrusions are observed, shows no evidence for erosion and uplift within the Springar Formation. At this location, all the points in Fig. 13d are plotted below the Storvoll’s trend line. Nevertheless, the Naust Formation reveals mild uplift of about 100 m and net erosion of up to 200 m (Fig. 12d).

## Spatio-temporal distribution of erosional features along the basal shear zones of the slides

**Figure 14:**
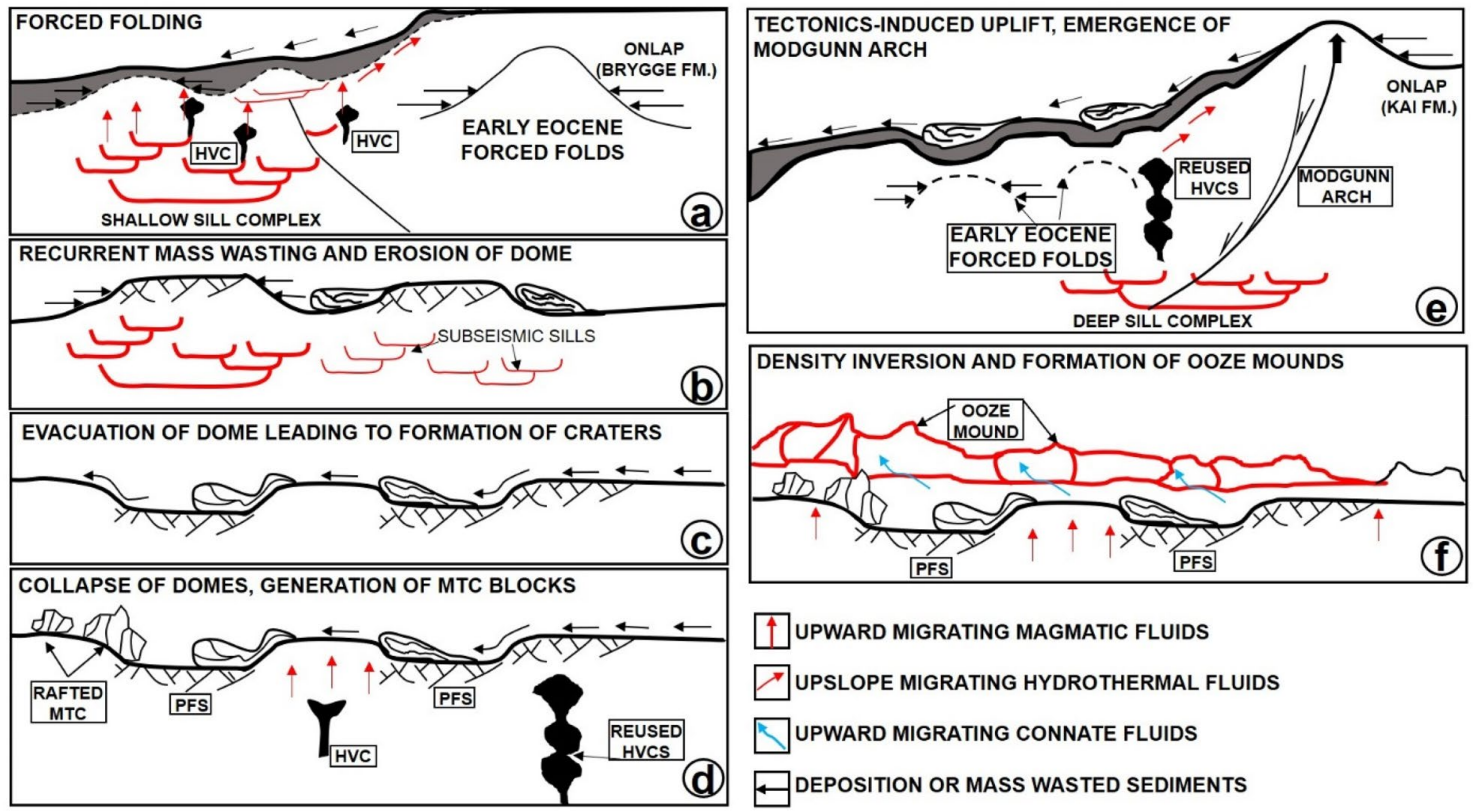
Conceptual model showing the evolution of craters in relation to magmatic emplacement, overburden uplift and mass wasting in the study area. Since the craters were preferentially formed on paleo-highs, their evolution is strongly tied to repeated erosion of these paleo-highs during recurrent mass wasting in the study area.

Here, we describe the inlets and terraces associated with craters as erosional features, despite their affinity with subsurface structures such as faults. Their distal lobate to rounded geometries signify that they are erosional inlets formed gradually, rather than features derived from short-lived or instantaneous crater collapse. We further confirm that the sidewalls imaged on the flanks of the terraces are non-tectonic irrespectively of their close interaction with sub-surface structures and polygonal faults. The sidewalls are simply erosional boundaries of the craters developed during sediment evacuation (see also^41–44^). Such an interpretation agrees with data in^13^ and^14^. In parallel, tectonic, and polygonal faults under the basal shear zone of MTC X are pre-depositional in origin and existed prior to the downslope translation of this mass-wasting deposit. As for the craters, their spatial distribution and location relative to the magmatic intrusions reveal they were preferentially formed on mechanically incompetent paleo-highs. These paleo-highs were induced by the forceful emplacement of underlying magmatic sills, which generated forced folds and were the foci of vertical fluid flow since the Early Eocene (Fig. 14). Mechanical weakness on these paleo-highs is primarily related to the following mechanisms: (a) localized forced folding, (b) repeated fluidization of the overburden and (c) mass wasting.

Forced folding in overburden rocks has been documented in multiple geologic settings as a direct manifestation of magma intrusion, and associated overburden uplift can reach several hundreds of meters: ~ 350 m in the NE Rockall Basin^37^, ~ 210 m in Southern Australia^38^, ~ 346 m in Ethiopia Alu Dome^45^, ~ 296 m in the Irish Rockall Basin^46^, ~ 171 m in Stappen High^47^. Forced folds also record an amplitude of ~ 780 km^2^ in the Vøring Basin, where overburden strata are underlain by interconnected sill complexes^48^. Therefore, the occurrence of magmatic sills below craters C1, C2, C5, C6 and C7 is not a coincidence, rather a consequence of local uplift due to magmatic sill emplacement. Here, we stress the use of the term ‘local uplift’ as there is also evidence for regional uplift at the level of the Springar Formation (Fig. 12a,e). The onlapping seismic reflections in Fig. 12e indicate the study area was uplifted during the deposition of the Kai and the youngest Brygge formations. However, it is unclear if regional uplift reflects the presence of wider-than-imaged magmatic sill complexes, or the effect of other more regional tectonic events^16^. Nevertheless, the 1-D uplift estimates in this work prove that the Springar, Tare, and Tang Formations were uplifted due to the intrusion of magmatic rocks in the study area.

Striking evidence for focused fluid flow includes hydrothermal vent complexes, polygonal faults within the Kai and Brygge Formations, and the high-amplitude packages below Horizon H1 that mark the presence of a fossilized opal A/CT boundary^49^ (Fig. 2). All these features constitute valid evidence for past episodes of fluid flow and associated diagenesis. Hydrothermal vent complexes are widespread in the study area and related to Eocene magmatism (Figs. 2, 11b and 12d). A number of these vent complexes terminate in strata of the Springar to Tare formations (Figs. 2 and 12) and were possibly reutilized for fluid plumbing after their formation^17^. Depending on the residence time of the magma feeding the sills, some of the magma migration pathways can remain active several years after the first episode of magma intrusion, leading to formation of more hydrothermal vents and resulting migration of fluid in strata^50,51^. Hence, it is likely that fluid was still fed into the overburden during the translation of the interpreted MTCs and ooze mounds. Svensen et al. (2003) have shown that vent complexes in the Vøring Basin can remain active some 50 Ma after the emplacement of their associated magmatic sills. Polygonal faults typically are formed during early burial upon compaction-related dewatering and further release of fluids in the subsurface^52^. Similarly, the opal A/CT boundary is a diagenetic front formed during burial, at specific temperatures and pressure conditions. It caused the dissolution of opal-A in silica-rich sediments and its precipitation as opal-CT, a process usually associated with the production of fluid^36,53^. The combined effect of fluid sourced from the hydrothermal vents, and other secondary sources such as polygonal faults and the opal A/CT boundary, likely elevated pore fluid pressure and compromised the integrity of the paleo-highs (forced folds) upon which the craters later developed.

## A conceptual model explaining the development of giant craters on the Mid-Norwegian margin

We show the intrusion of magmatic sill complexes in the Storegga and Modgunn Arch areas to result in local uplift of supra-sill strata and their forced folding (Fig. 14a). Subsequently, hydrothermal vents were formed by the release of volatile fluids and gases generated in the metamorphic aureoles of the sill^54–56^. Some of these vents may have remained active several million years after the first intrusion of this magma and were likely reutilized, plumbing fluid into the overburden strata^17^. Hence, fluid migrating upward through vents and other conduits interacted with fluid trapped near polygonal faults and that released during the transformation of opal-A into opal-CT. The combined action of these distinct fluids weakened the continental slope, especially locally uplifted areas above the sills, which became more susceptible to collapse or failure (Fig. 14b). In addition, the forced folds were the loci of enhanced faulting (Figs. 12 and 14b), which potentially influenced fluid migration in overburden strata^57^.

Fluids migrating from deep sources reduced the shear strength of continental-slope strata. Recurrent mass wasting during the downslope translation of MTC X resulted in the selective cannibalization of mechanically incompetent areas (Fig. 14c,d). Uplifted areas were excavated to form craters. In addition, slide blocks imaged on the seismic profiles are interpreted to have been locally sourced from the collapse of uplifted areas (Fig. 14c,d). Recurrent mass wasting and the translation of younger MTCs on the continental slope was further enhanced by the regional uplift that led to the formation of the Modgunn Arch in the Miocene (Fig. 14e). Sediment oozes were subsequently deposited above MTC X (Fig. 14f) possibly in response to further mass wasting and continuous fluid discharge from the subsurface (Fig. 14f). Alternatively, we propose that once the downslope translation of the failed mass stopped, density differences between the sediment oozes and the material in MTC X triggered the migration of ooze to the top of this latter MTC to form ooze mounds (see^13^ and^14,58^). It is also likely that the ooze mounds are younger mass-transport complexes formed above the craters, similar to those on the Utgard High (see^59^). In a further episode of basin structuring, the basal shear zones of younger MTCs (such as the Storegga Slide) were influenced by the inherited (and rugged) topography formed by the ooze mounds and craters (Figs. 5b, 6a,b and 7b). This is clear from the presence of a younger crater and terraces at the seafloor in the SMA area, for instance (Fig. 4a).

## Wider implications for understanding formation mechanism of craters along continental margins

Formation mechanisms proposed for previously studied craters include seismic shaking^4,5,10,60^, the reactivation of faults^3^, localized erosion and sediment progradation^3^, erosion by bottom or turbidity currents^1,2^, tectonics^5,8^, density inversion promoted by sediment load^14^, and subsurface fluid migration^5^. In this study, we have uniquely shown that all these processes may have had a role in the formation of giant craters and, most importantly, show a cause-effect relationship between magmatic intrusions, prolonged subsurface fluid flow, and the formation of giant craters. As opposed to previous models where the impact of magmatism was never or scarcely reported, we show that prolonged magmatic activity such as overburden uplift and hydrothermal venting can primarily weaken and pre-dispose a continental slope to failure, thus leading to the selective cannibalization of the seafloor and the formation of giant craters. The effect of tectonics, diagenesis and density inversion are highlighted as secondary formation mechanisms.

## Conclusions

This study used 2-D, 3-D seismic reflection and well data from the Storegga and Modgunn Arch areas, Møre and Vøring basins, offshore Norway, to characterize the seismic expression, scale, geometry, and evolution of giant craters at the basal shear zone of a mass-transport complex. A conceptual model was developed to explain the causal effect between the location of craters and regions of forced folding in supra-sill strata. We demonstrated the existence of several paleo-highs in the study area prior to the translation of a mass-transport complex (MTC X). Our findings show that most craters are associated with a seismic horizon correlating with the basal shear zone of MTC X. Importantly, we show that the primary factor enabling the development of giant craters was forced folding due to magmatic emplacement. The close spatial connection between the craters and the underlying network of sill complexes implies that intrusive rocks along the NE Atlantic margin play a critical role in shaping the basal configuration of MTCs. The model presented here further justifies the importance of subsurface fluid plumbing as a major control on mass wasting along continental margins.

## Data and methods

### Interpreted dataset

The primary data for this study include 3 three-dimensional (3-D) seismic reflection volumes and several regionally tied two-dimensional (2-D) seismic profiles (Fig. 1a). The interpreted 3-D seismic data include the MC3D-MGS2002-FULL-OFFSET_3D_FM_TVFGC (South Modgunn Arch, SMA), MC3D-RHD99_3D_FM_TVFGC (Havsule), NH0003-FULL_3D_FM_TVFGC (Solsikke) surveys. All the seismic data are time-migrated, zero-phased at the seafloor reflector, with a vertical scale and sampling rates of 8 s and 4 ms, respectively. The seismic datasets are displayed in the European or reverse SEG (Society of Exploration Geophysicists) polarity convention, implying that a downward increase in acoustic impedance, or trough, is shown as a red reflection, while decreases in acoustic impedance or peaks are shown in blue. The overall quality of the seismic cubes is good, with a dominant frequency spectrum that ranges from approximately 40 Hz to 60 Hz for the intervals of interest. Hence, the dominant wavelength of 37 m to 55 m with velocity of 2200 m/s gives vertical seismic resolutions or ‘limit of separability’ of ~ 9.16 m to ~ 13.75 m at λ/4, one quarter of the dominant wavelet^61,62^. The 2D seismic lines are generally oriented in a NNE-SSW, NW–SE, or ENE-WSW direction, are irregularly spaced, and have a recording length of 10,000 to 12,000 ms TWT. The 2D seismic lines were used for regional correlations between key seismic-stratigraphic horizons, mapping of the top Storegga Slide, and in the identification of magmatic sills. Additionally, four wells (6302/6-1, 6403/6-1, 6403/10-1 and 6404/11-1) were made available for the purposes of this study (Fig. 1a). The wells contain check-shot and conventional wireline logs such as gamma ray, density, sonic and neutron. These data were complemented by information on the ages of the seismic units, their lithology, and accurate seismic-stratigraphic correlations across the three seismic surveys.

### Seismic interpretation

Four horizons were mapped and later tied to three of the wells in the study area (Figs. 1c, 2, 4 and 5). These horizons include: (a) H1, the top of the Brygge Formation (c) H2, a seismic reflection in the interior of the Kai Formation correlating with the top of the ooze interval within the Kai Formation (Riis et al. 2005), and (d) H3 and H4, which are part of the Naust Formation (Figs. 1c, 2 and 3). The mass-transport complex (MTC X) in the study area was defined using pre-existing tectonic stratigraphic information from previous workers^31,63^. On seismic profiles, the upper surface of MTC X is a rugged to ridged surface located above chaotic to moderately deformed seismic reflections of variable amplitude^64^. Conversely the basal shear zones of MTC X—comprising prominent m-size zones with a mélange of reworked strata and clasts, ripped blocks of seafloor material and faulted near-seafloor material—separate their internal, disrupted strata from relatively continuous deposits underneath^65,66^. To support the interpretation of the basal shear zone of MTC X and internal fill of the craters, variance maps were used to map striations and other basal kinematic indicators below MTC X. The variance attribute is a seismic time-derived attribute that measures the ‘dissimilarity’ between seismic traces and converts a volume of continuity into a volume of discontinuity, highlighting structural and stratigraphic boundaries^67^. On variance maps, MTC X is shown as an interval comprising chaotic reflections with well-defined basal shear zone upon which the failed masses of sediment were translated^64^. Variance maps are also good discriminator of lithology. Chaotic seismic facies (interpreted as debris flow deposits) are shown as intervals with low variance when compared to the well-bedded high- to medium-amplitude facies of underlying, undeformed strata. In addition, faults represent trace-to-trace variability and are shown as features with high variance coefficients. Furthermore, the interpretation of magmatic sills relied on identifying their typical seismic amplitude, geometries, and lateral continuity within the host-rock strata. The magmatic sills are characterized by their abrupt, localized brightening of positive-amplitude reflections with partial or complete loops of ‘peak-trough-peak’ reflections that are similar to the seafloor reflection^68^. As magmatic sills have greater densities and seismic velocities when compared to their surrounding strata, these are responsible for high acoustic-impedance contrasts at the sill-host rock contacts^69^.

## One-dimensional (1-D) estimates of magma-related uplift

In addition to the workflow for the seismic interpretation, we computed one-dimensional (1-D) models based on sonic (p-wave velocity) and gamma-ray data from wireline logs to obtain an overview of net erosion and uplift in the study area (Figure A1). Magma-related uplift was estimated using a normal compaction (representing no uplift) or reference velocity-depth trend (see blue line in Figure A1b). The red line above this latter is a velocity-depth trend of an area that has undergone uplift and erosion. Since eroded rocks are still compacted to a similar degree to what they were at their deepest burial point, the red line therefore plots higher than the blue reference trend (Figure A1b). The difference in depth between the reference trend and the eroded rock trend corresponds to the vertical difference between present day burial depth and the maximum burial depth^70^.

The reference trend line used in this work was developed by^70^ based on a simplified linear velocity-depth trend. The trend line is expressed as *Z* = 1.76*Vp*–2600, where Z is depth in meters and Vp is the p-wave velocity in meters per second. To use the Storvoll’s trend line, three basic assumptions were made: (a) the velocity-depth trends are linear. Normally, velocity increases with depth, and this is the case for the Norwegian Sea, though velocity trends may vary with depth^71^. A linear relationship was assumed for the relatively short intervals considered in this study; (b) lithology is assumed to be homogeneous, although large variations in lithology and facies are likely to occur in the study area; and (c) the thermal history across the entire area is assumed to be uniform. The Storvoll’s velocity trend approaches zero (Figure A1c), hence it provides the best trend in comparison to the velocity trends of^72–76^. Furthermore, the effect of varying pore-fluid saturations on the sonic velocity is low in the study area since the wells contain pore water and not hydrocarbons^77^. In this study, the reference trend has been allocated an uncertainty value of ± 200 m^70^ in the North Sea. The p-wave velocities used to compute velocity-depth trends were calculated from compressional sonic data. Hence, the p-wave velocities (m/s) are converted from acoustic slowness (μs/ft.).

To ensure the consistency of our model, all data points selected for analysis were taken from the Naust Formation to the total depth (TD) in the wells. Hence, the stratigraphic units analyzed in our uplift models include the Lysing, Kvitnos Nise, Springar, Tang, Tare, Kai, and Naust formations. Special focus was put on the Springar Formation, which is most affected by magmatic intrusions. For the velocity-depth analyses, shale lithologies were chosen for their consistency with Storvoll’s reference trend (Figure A1d). Strata with a clay volume greater than 80% were considered as clean shales for the data analysis. An advantage of using shaley lithologies is that thin cracks in their interior would not affect the velocities relative to sandy lithologies^70^. Volume of clay (Vcl) was calculated from the gamma-ray logs using the equation Vclay (Vcl) = GRmax- GRmin / GRlog- GRmin^78^, where GRmax is the 100% clay limit or line, GRmin is the 100% sand limit or line, and GRlog is the value of the log for a particular data point (Figure A1d). Minimum and maximum gamma ray values were manually selected for each well log. At certain depths, the gamma-ray log varied dramatically due to changes in casing size, as also indicated by a change in caliper log on the available wireline data. Where this occurred, multiple intervals were taken from the gamma-ray log to adjust the minimum and maximum values selected.

## Supplementary Information

Supplementary Information.

## Data Availability

The data that support the findings are available from the Norwegian University of Science and Technology (NTNU) or DISKOS. Restrictions apply to the availability of these data, which were used under license for this study. Data are available with permission of NTNU.

## Code availability

MATLAB codes used here can be provided upon request from Professor Kenneth Duffaut.
