# Supplementary material for: Giant paleo-seafloor craters and mass wasting associated with magma-induced uplift of the upper crust
Source: Sci Rep. 2022 Mar 15;12:4392. doi: 10.1038/s41598-022-08205-0 (PMC8924249; doi:10.1038/s41598-022-08205-0)
Supplement: Supplementary file 1 — Supplementary Information. [file 41598_2022_8205_MOESM1_ESM.docx]

**Giant paleo-seafloor craters and mass-wasting associated with magma-induced uplift of the upper crust.**

Omosanya, K.O^1^; Duffaut, K^2^, Alves, T.M^3^., Eruteya, O.E^4^., Johansen, S.E^2^, Waldmann, N.^5^

^1^Oasisgeokonsult, 7052. Trondheim. Norway.

^2^Department of Geoscience and Petroleum, Norwegian University of Science and Technology, Norway

^3^3D Seismic Lab, School of Earth and Environmental Sciences, Cardiff University, Main Building, Park Place, Cardiff, CF10 3AT, United Kingdom.

^4^Geo-Energy/Reservoir Geology and Basin Analysis Group, Department of Earth Sciences, University of Geneva. Switzerland.

^5^Dr Moses Strauss Department of Marine Geosciences, University of Haifa. Israel.

Corresponding author:

[kamal.omosanya@oasisgeokonsult.com](about:blank)

ORCID: 0000-0001-8959-2329

Appendix


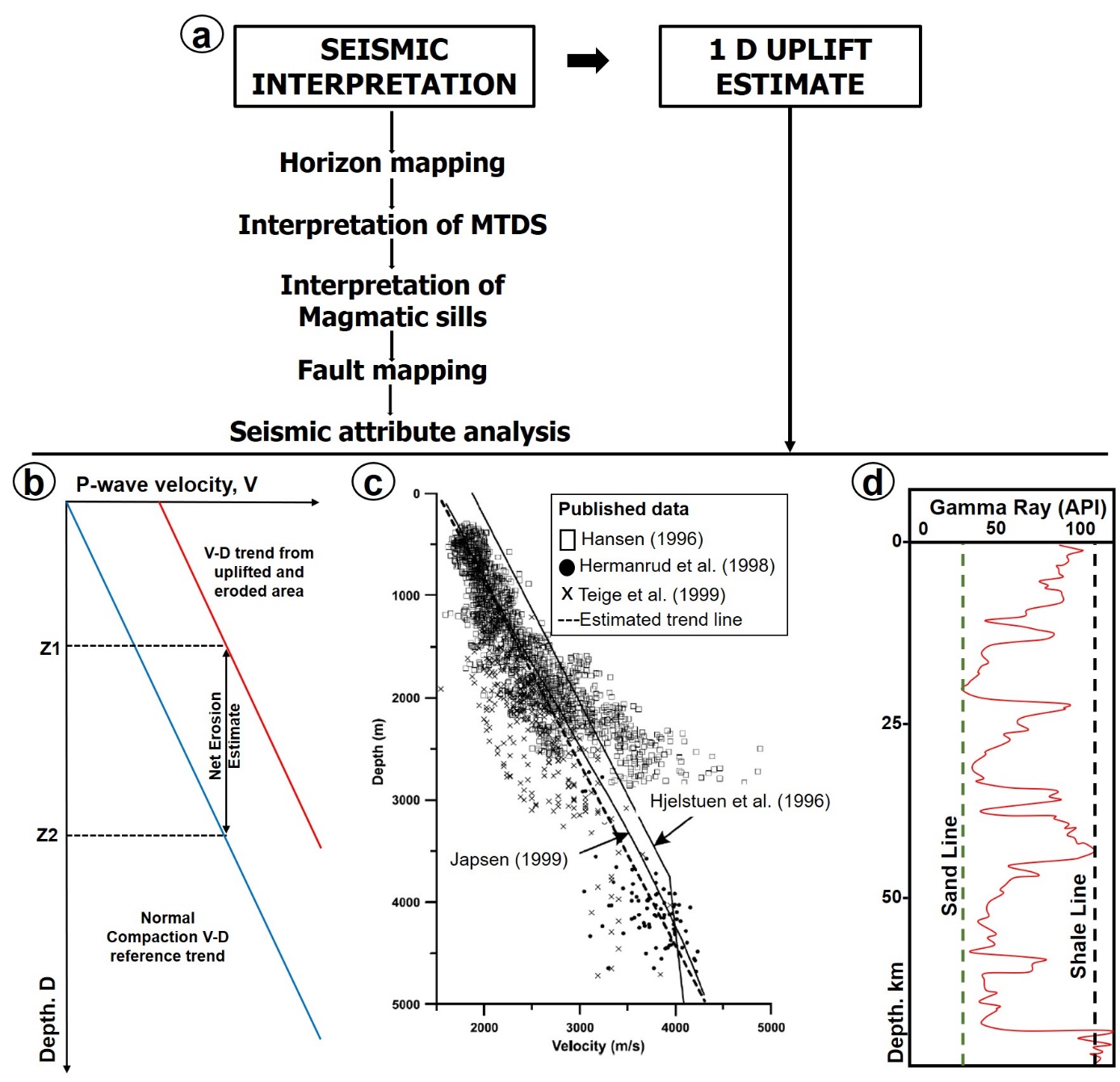


Figure A1: (a) Flow chart showing the methods used in this study. These include seismic interpretation, and 1-D uplift estimate for understanding the influence of magmatic intrusions on overburden uplift in the areas where craters are observed. (b) to (d) Step-by-step process including data quality control and estimation of uplift using a best fit line for the plot of P-wave velocity with depth. In (b), the difference between the reference trend (red) and the blue trend line, which has undergone uplift and erosion will give the net erosion estimate. The net erosion is the difference between present day burial depth for the formation (Z1) and maximum burial depth (Z2).


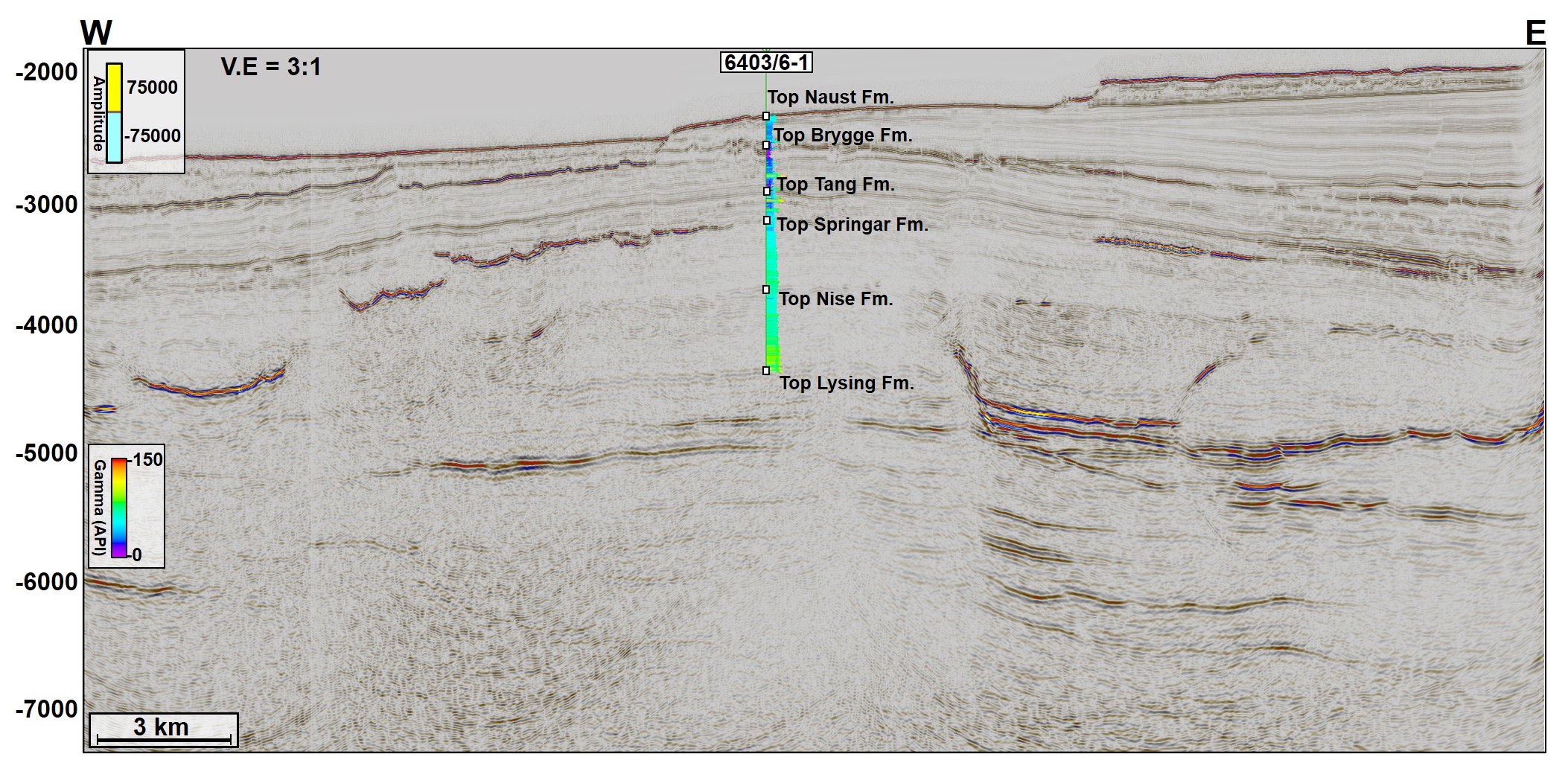


Figure A2. Uninterpreted seismic profile of Figure 2


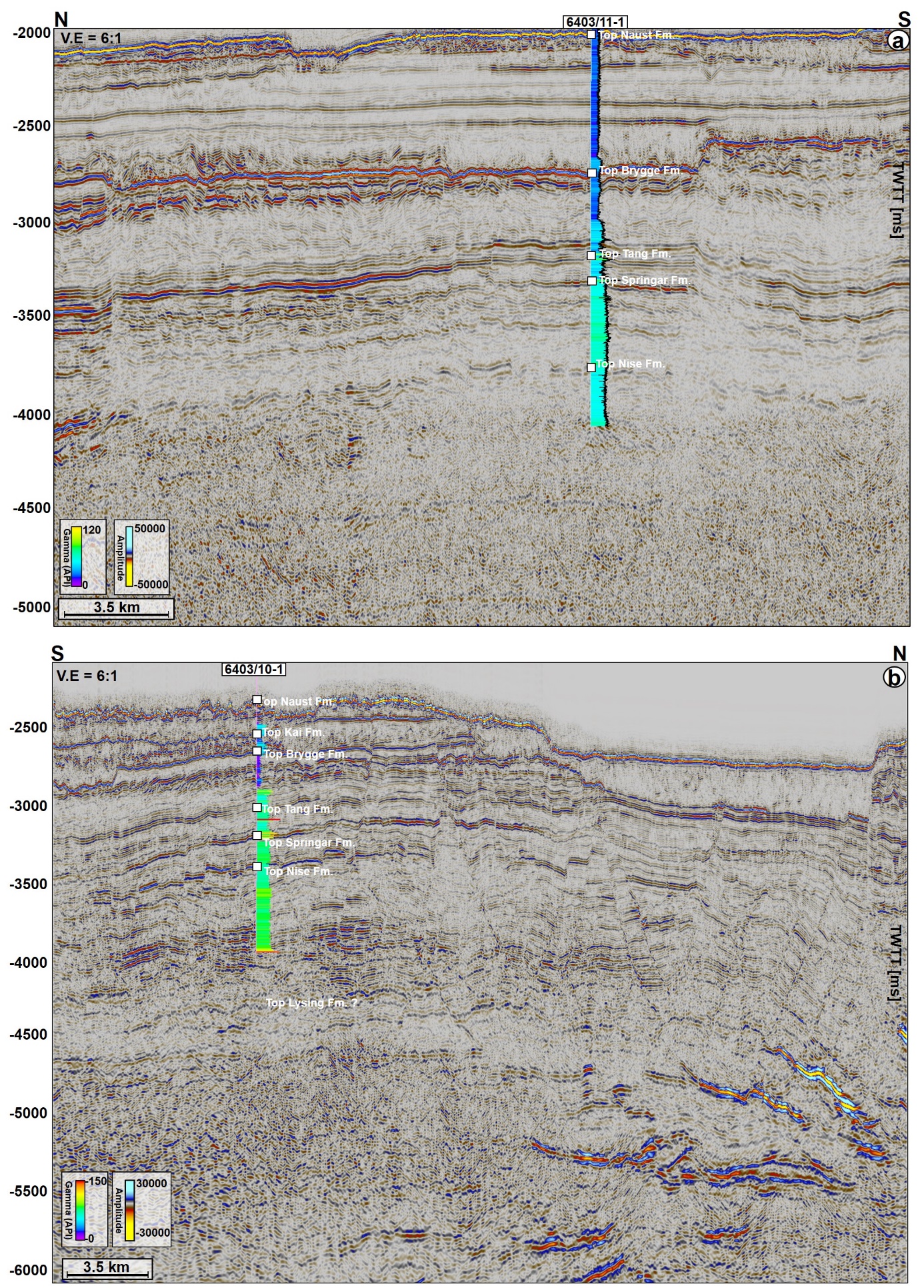


Figure A3. Uninterpreted seismic profile of Figure 3.


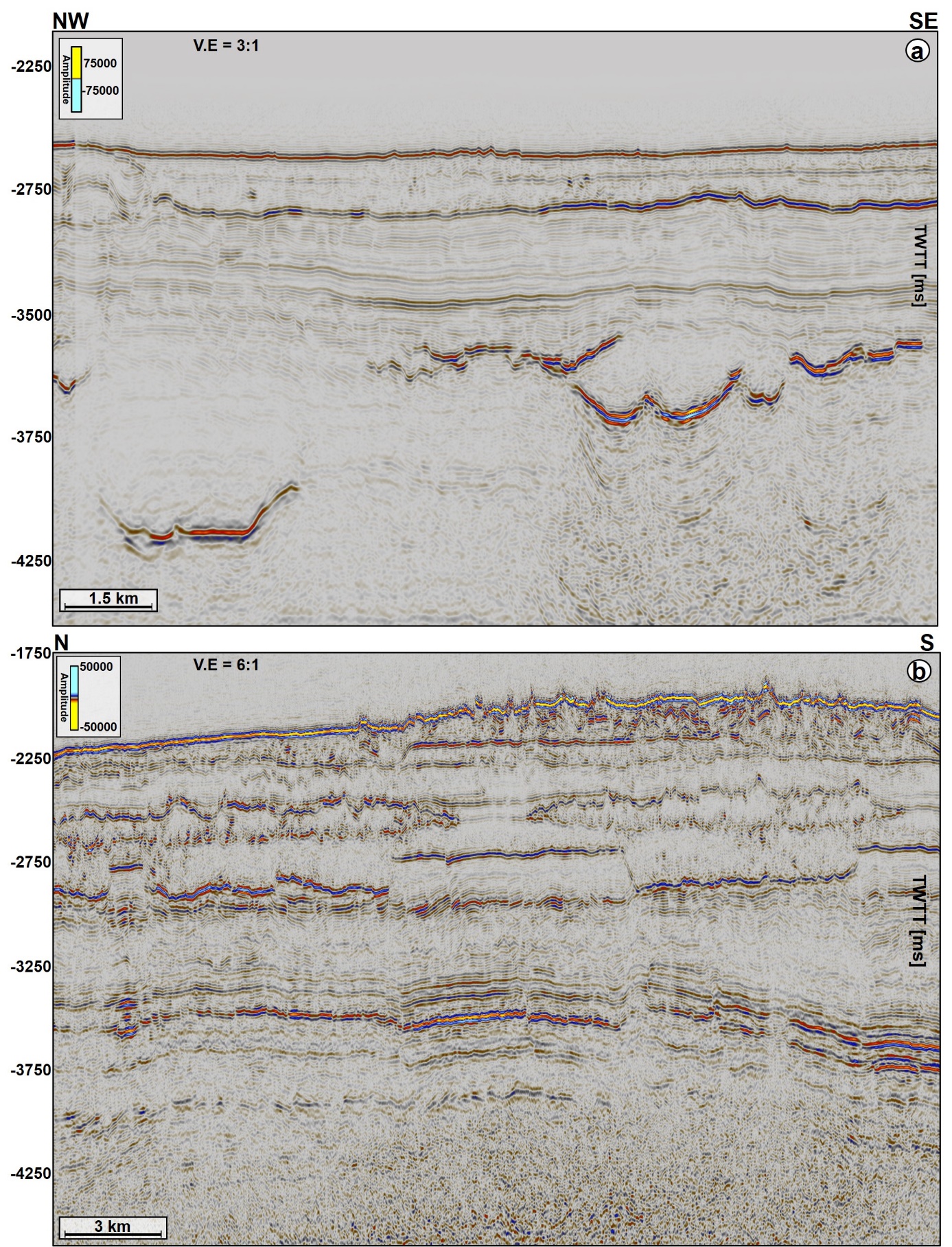


Figure A4. Uninterpreted seismic profile of Figure 5


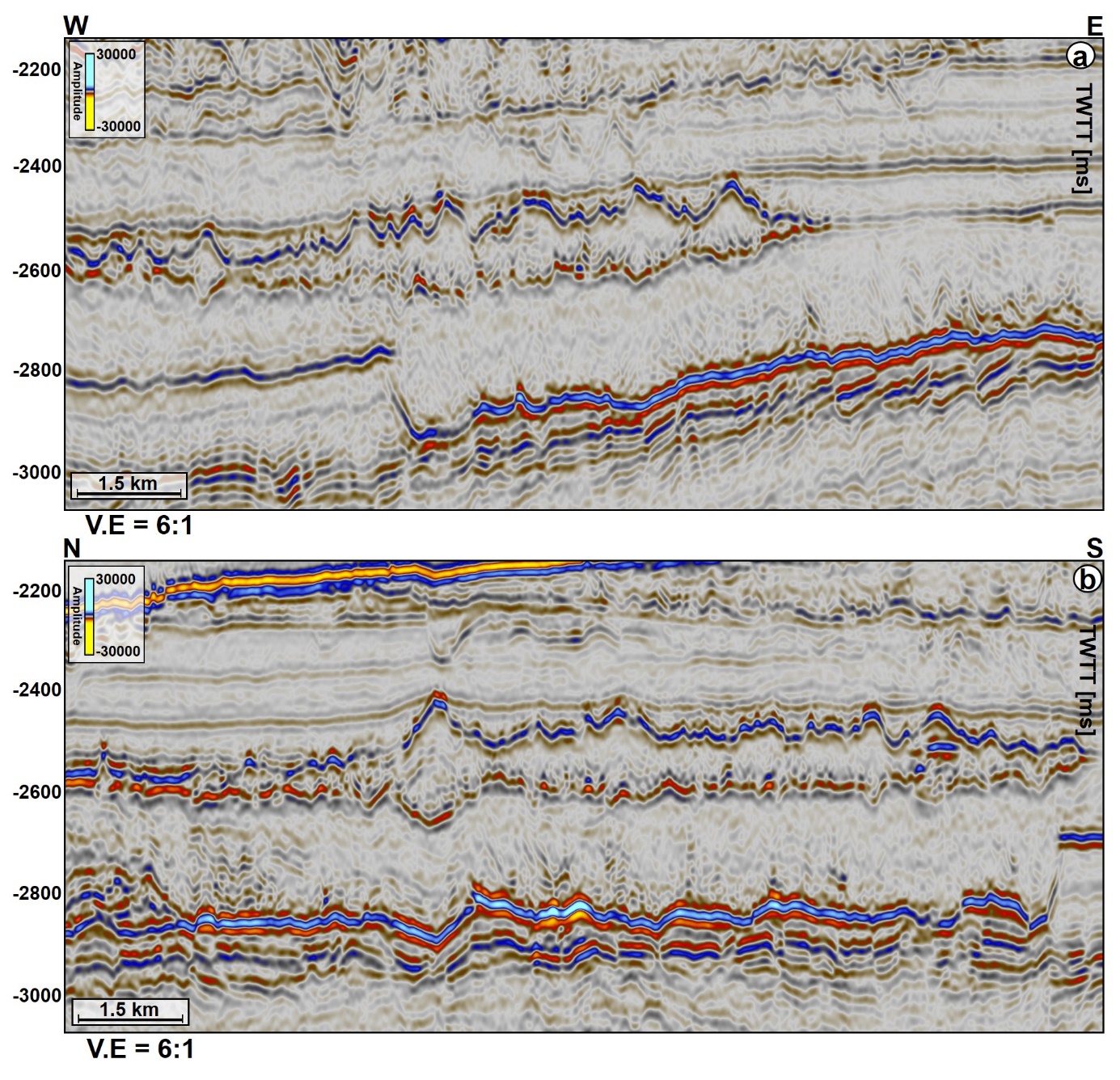


Figure A5. Uninterpreted seismic profile of Figure 6


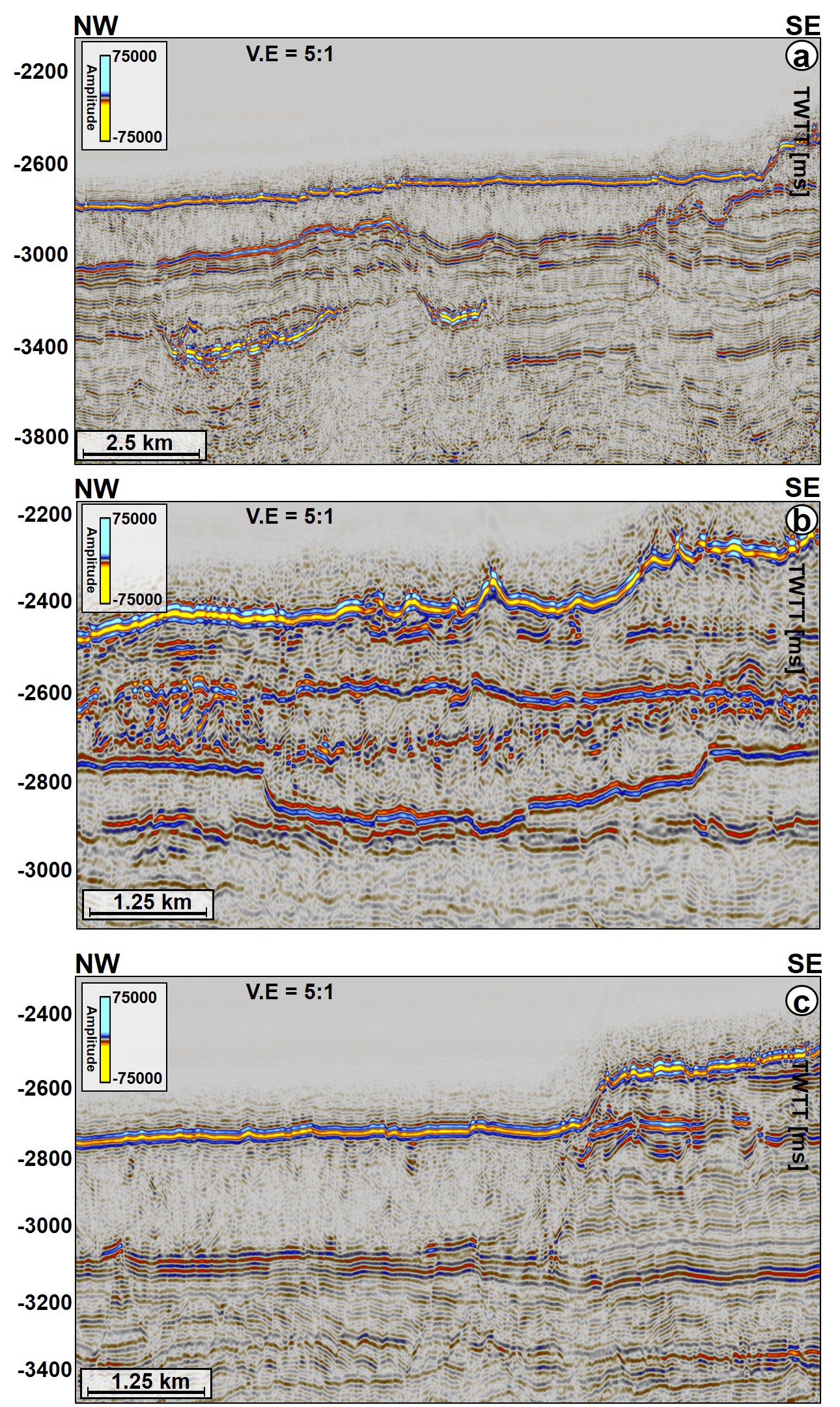


Figure A6. Uninterpreted seismic profile of Figure 7


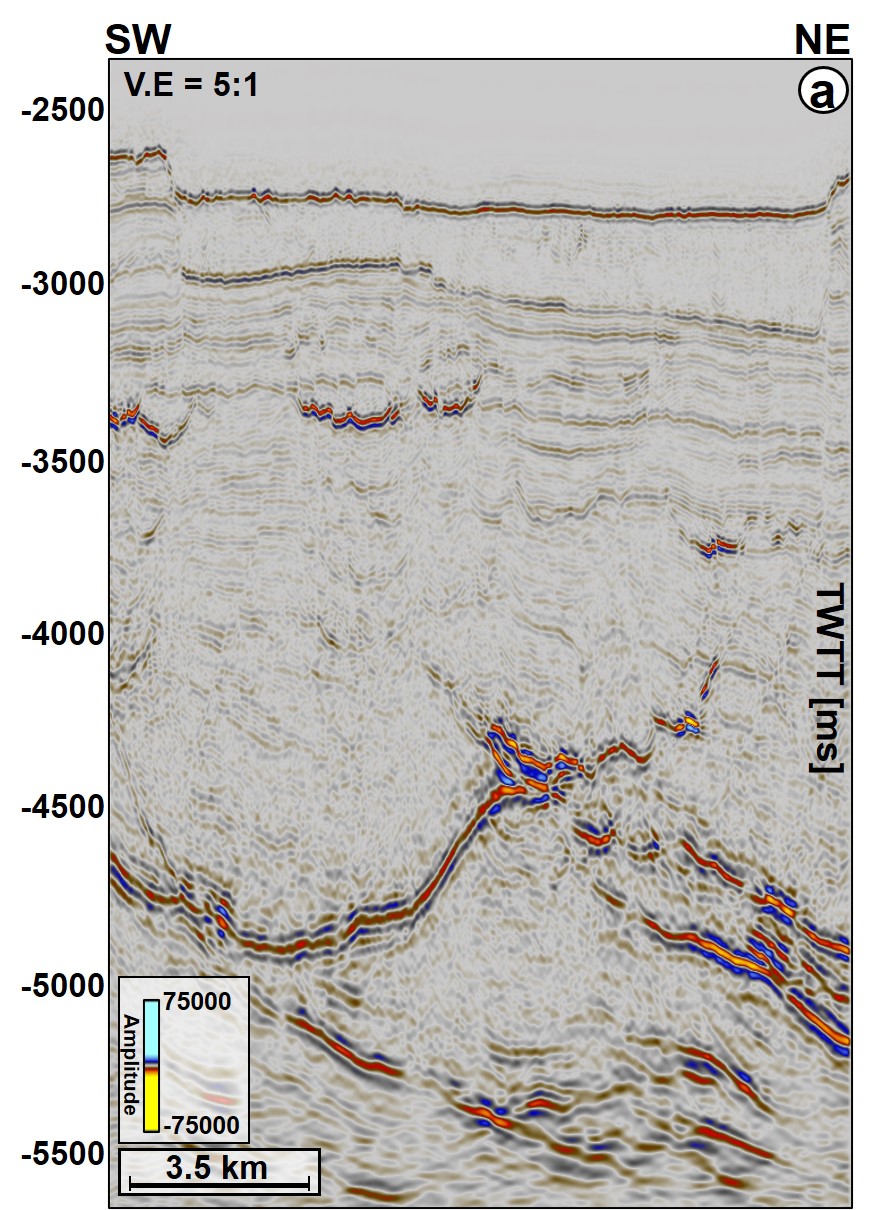


Figure A7. Uninterpreted seismic profile of Figure 9


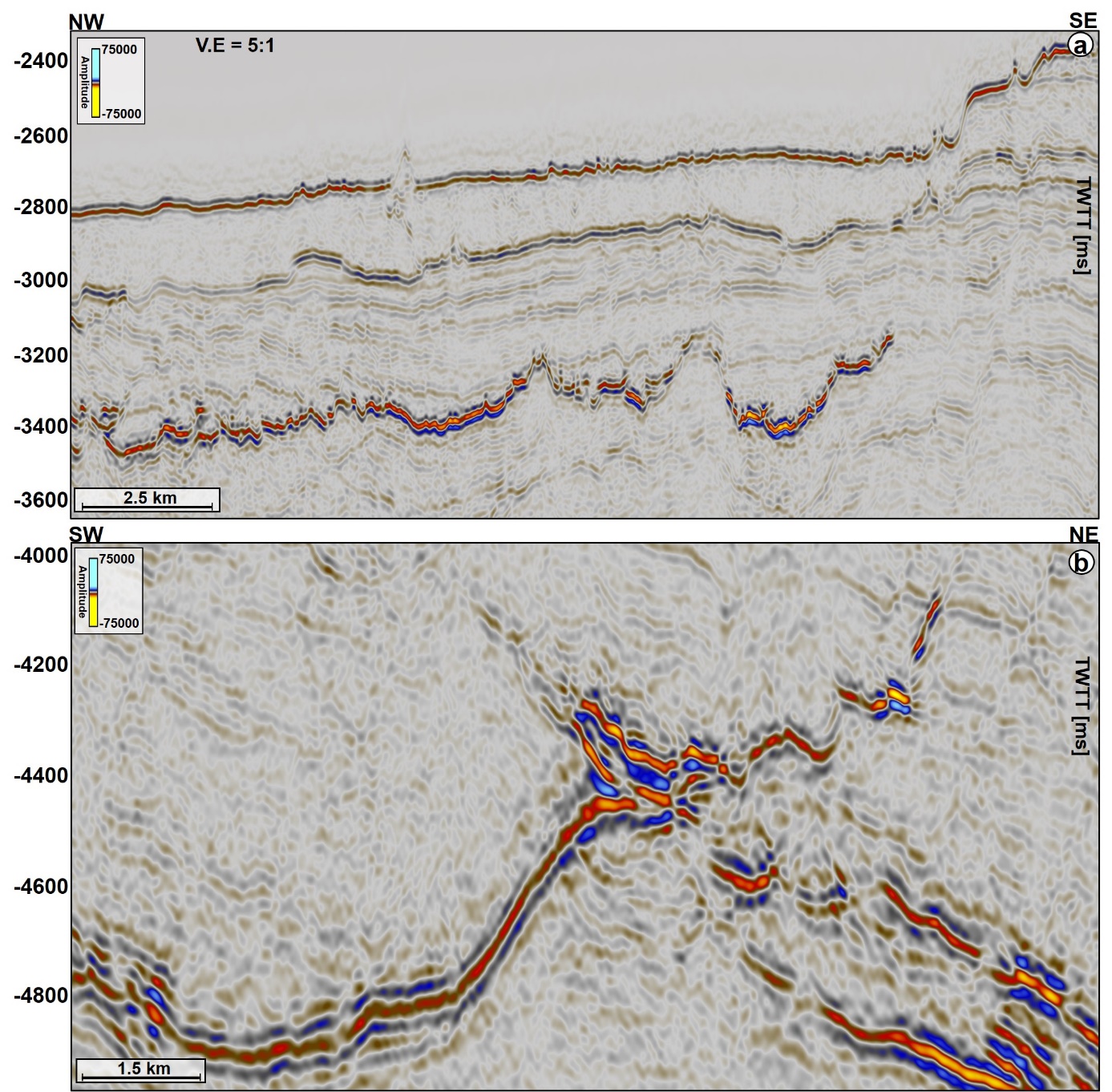


Figure A8. Uninterpreted seismic profile of Figure 10


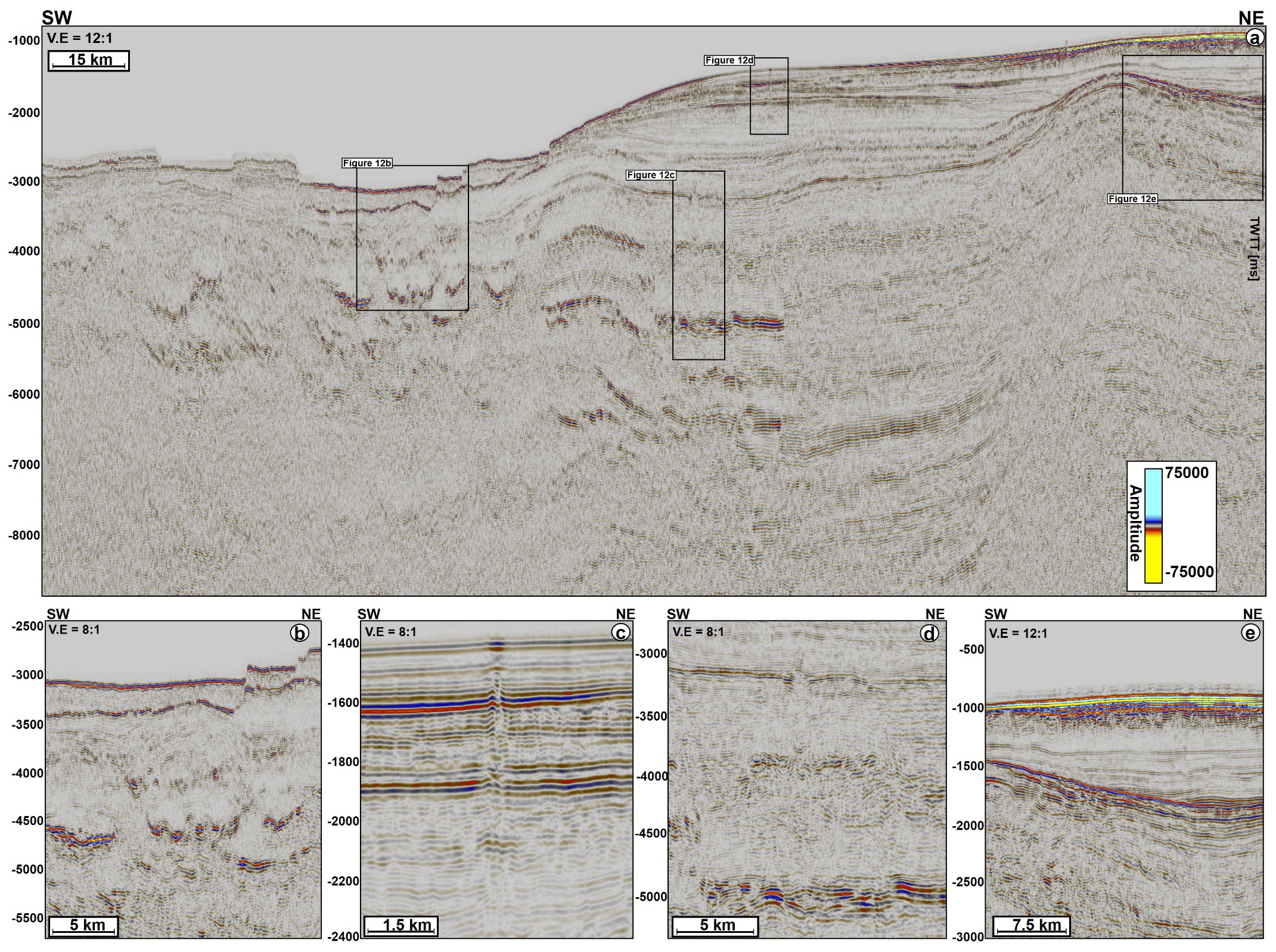


Figure A9. Uninterpreted seismic profile of Figure 12
